# Flora-YOLO: A lightweight framework for real-time rose grading on edge devices via structural optimization and feature refinement

**DOI:** 10.1371/journal.pone.0358742

**Published:** 2026-09-21

**Authors:** Wenwei Liu, Jinyu Xu, Guoao Wang, Shuo Ding, Yuanbo Zhang, Runze Tian, Zejiang Li, Yuanzhe Ji, Li Zhang

**Affiliations:** 1 School of Airspace Science and Engineering, Shandong University, Weihai, Shandong, China; 2 School of Mathematics and Statistics, Shandong University, Weihai, Shandong, China; 3 Shandong Key Laboratory of Intelligent Electronic Packaging Testing and Application, Shandong University, Weihai, Shandong, China; Indian Institute of Technology Kharagpur, INDIA

## Abstract

Precise flower grading in modern agriculture is critical for determining market value but remains hindered by inefficient manual labor and the computational constraints of edge devices. To address these challenges, this paper introduces Flora-YOLO, a lightweight and robust detection framework built upon the YOLO11n architecture. To overcome the inherent bottlenecks of agricultural vision, the framework incorporates four targeted optimizations, explicitly distinguishing novel components from adapted architectures: (1) an adapted C3k2-based Sandglass-Gated Bottleneck (C3k2_SGB) module to extract and amplify minute floral textures; (2) an integrated Adaptive Downsampling (ADown) module to preserve critical high-frequency spatial cues under severe foliage occlusion; (3) a newly proposed CSP-based Variance-Gated Attention (C2VGA) hybrid attention mechanism to amplify the weak semantic signals of early-stage buds while suppressing background noise; and (4) a novel Focused Shape-Intersection over Union (FSIoU) loss function to provide shape-aware gradient guidance for the precise bounding-box regression of irregular, non-rigid floral boundaries. Evaluated via 5-fold cross-validation on a custom dataset, the model achieves a stable 86.7% mAP@0.5 and 50.4% mAP@0.5:0.95. This represents a 3.4% accuracy improvement over the baseline, achieved alongside a 28.6% reduction in computational load, requiring only 1.73 M parameters and 4.5 GFLOPs. Furthermore, out-of-domain testing and evaluation on public agricultural datasets confirm the model’s cross-species generalization. Finally, hardware deployment on both Android terminals and an NPU-accelerated NXP i.MX 8M Plus platform yields a real-time inference speed of 11.05 FPS with a 90.5 ms latency. These results establish Flora-YOLO as a highly efficient, accurate, and scalable solution for automated, edge-based sorting in precision floriculture.

## 1. Introduction

As a cornerstone of ornamental horticulture, the global flower industry necessitates the refined cultivation of high-value crops like roses [1]. Precise grading of rose phenological stages—from initial bud to full bloom—is critical for determining commercial value and optimizing production decisions such as harvest timing and pest control [2–4]. Traditionally, this task relies on manual visual observation, which is labor-intensive, subjective, and difficult to scale for large-scale commercial production [5].

Recent advancements in deep learning have shifted the paradigm toward automated plant phenotype analysis [6]. While Convolutional Neural Networks (CNNs), such as ResNet50, have achieved over 97% accuracy in flower species identification [7–9], these classification-oriented models struggle with practical grading tasks. Their primary limitation lies in their design objective: they excel at differentiating species but fail to discern subtle, intra-species phenological variations required for maturity assessment [10,11].

To overcome these constraints, research has transitioned toward object detection to localize and identify multiple instances simultaneously. Two-stage detectors like Faster R-CNN and Mask R-CNN have been applied to flower phenology with high accuracy [12–14]. However, their “proposal-then-classification” paradigm imposes significant computational overhead and slow inference speeds. Moreover, they often exhibit suboptimal performance on small or densely distributed targets, rendering them ill-suited for real-time deployment on resource-constrained agricultural edge platforms [15,16].

Concurrently, Transformer-based architectures like DETR and Swin Transformer have emerged, theoretically surpassing CNNs by modeling long-range dependencies [17]. However, their practical application in agriculture faces critical bottlenecks. First, the quadratic scaling of self-attention mechanisms imposes prohibitive computational and memory overheads, especially when processing the high-resolution inputs required for small-target detection on edge devices [18,19]. Second, global attention can paradoxically dilute focus on localized features of small targets, as attention maps often become overly diffuse amidst complex agricultural backgrounds [20]. This suggests that for phenological grading, a guided, efficient attention mechanism integrated with a streamlined CNN backbone and rich feature representation [21] may be more effective for practical deployment than pure global attention.

The YOLO (You Only Look Once) series has consistently advanced the Pareto frontier of object detection, with the YOLO11n variant offering an optimal balance of feature extraction and a minimal parameter footprint [22]. Current research in YOLO-based flower detection generally follows two divergent paths. One branch prioritizes maximal accuracy by integrating complex modules, such as SE attention [23], Coordinate Attention (CA) [24], or hybrid state-space models like ROSE-MAMBA-YOLO [25]. However, these enhancements often lead to a 2.5-fold increase in parameters, posing significant deployment challenges on power-constrained agricultural platforms [26]. Conversely, the second branch emphasizes model efficiency for edge devices like UAVs, utilizing lightweight designs to reduce parameters and GFLOPs by up to 85% [27–29]. While highly efficient, such architectures often sacrifice discriminative power, struggling to distinguish between morphologically similar phenological stages. Consequently, practical agricultural deployment necessitates a new, task-specific equilibrium that maintains high grading precision without compromising real-time edge performance.

Despite these advancements, automated rose phenology grading in complex agricultural scenarios is still constrained by four primary physical and environmental bottlenecks. First, the fine-grained morphological ambiguity inherent to the task—characterized by high intra-class variance and low inter-class differences among blooming stages—makes texture extraction difficult for standard convolutions [30,31]. Second, ubiquitous foliage occlusion and dense clustering severely reduce visible features, causing critical spatial information to be lost during standard network downsampling [32]. Third, extreme small-target detection remains a persistent issue, as early-stage unbloomed buds occupy minimal pixel areas, causing their weak semantic signals to blend into the green background [33,34]. Finally, irregular morphologies and domain shifts, driven by fluctuating greenhouse lighting and varying camera perspectives, alter the perceived 2D geometry of flowers. This variability makes traditional rigid bounding-box regression highly inaccurate for non-rigid floral contours [35,36].

To systematically resolve these challenges, this paper introduces Flora-YOLO, an efficient detection framework built upon the YOLO11n architecture. The model incorporates four targeted structural optimizations, explicitly distinguishing novel contributions from domain-specific adaptations: (1) as an architectural adaptation, the C3k2-based Sandglass-Gated Bottleneck (C3k2_SGB) module is adapted to extract and amplify minute floral textures; (2) as a structural integration, the Adaptive Downsampling (ADown) module is integrated to preserve critical high-frequency spatial cues during spatial reduction; (3) as a novel contribution, the newly proposed CSP-based Variance-Gated Attention (C2VGA) hybrid attention mechanism is introduced to amplify the weak semantic signals of early-stage buds while suppressing background noise; and (4) as another novel contribution, the Focused Shape-Intersection over Union (FSIoU) loss function is formulated to provide shape-aware gradient guidance for the precise bounding-box regression of non-rigid boundaries.

The remainder of this manuscript is organized as follows. Section 2 details the construction of the custom dataset and introduces the methodological architecture of the Flora-YOLO model. Section 3 outlines the experimental design and evaluation metrics. Section 4 presents the experimental results, encompassing ablation studies, statistical validations, comparative analyses, real-world edge device deployments, and discussions. Finally, Section 5 concludes the paper.

## 2. Materials and methods

### 2.1. Dataset

#### 2.1.1. Data collection.

This study constructed a high-resolution image benchmark named the Mixed-Color Rose Dataset. The primary data was collected from July 10 to July 23, 2025, at the Shuangfu Flower Enterprise cultivation base in Dongying, Shandong, China. The dataset predominantly features the ‘Juice Balcony’ miniature rose—a staple commercial variety in regional protected horticulture, characterized by dense, multi-layered petals and vibrant orange-to-pink chromatic transitions. This focus ensures the dataset captures representative morphological and chromatic variations typical of large-scale commercial cultivation.

High-resolution smartphone cameras were utilized to simulate the optical sensors equipped on mobile agricultural robotics. Image acquisition was conducted under diverse greenhouse lighting (including direct sunlight and overcast conditions) and from multiple perspectives, with focal distances ranging from 20 to 50 cm. The primary baseline dataset consists of 1,002 high-resolution images, yielding exactly 8,474 precisely annotated floral instances. This dense instance-level distribution captures extreme scale variations and severe occlusion scenarios, providing substantial intra-class variance for robust feature representation. All images were resized to 640×640 pixels to align with the standard input requirements of the YOLO11 architecture.

To evaluate out-of-distribution (OOD) robustness in uncontrolled environments, an OOD Test Set consisting of 100 images (yielding an additional 1,327 instances) was constructed at a separate facility (Mingyang Flower Co., Ltd.). This set introduces significant environmental shifts, including differing greenhouse architectures, higher irrigation-induced humidity, and tighter crop spacing, which generate distinct background complexities and shadow patterns.

#### 2.1.2. Data annotation and statistical analysis.

**Fig 1:**
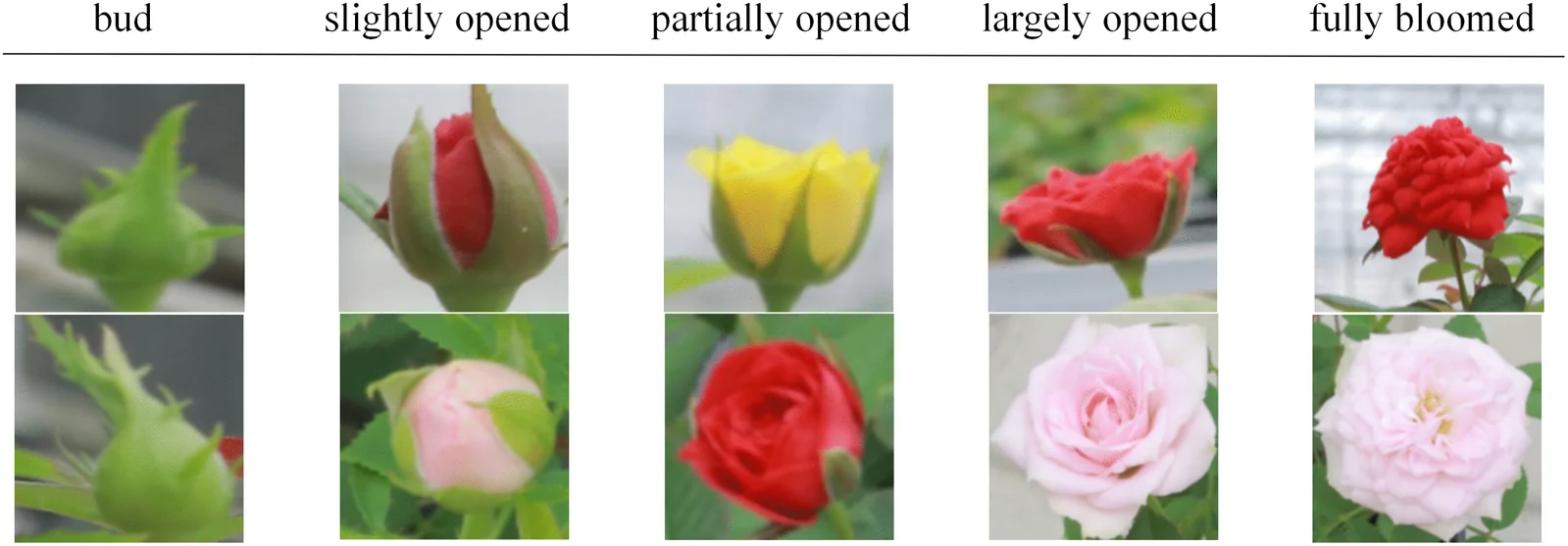
Representative samples of the five annotated phenological stages: bud, slightly opened, partially opened, largely opened, and fully bloomed.

A total of 1,102 images across both datasets were manually annotated using AnyLabeling. Following international commercial grading standards, floral instances were classified into five continuous phenological stages: Degree 0 (bud), Degree 1 (slightly opened), Degree 2 (partially opened), Degree 3 (largely opened), and Degree 4 (fully bloomed), as illustrated in Fig 1.

**Fig 2:**
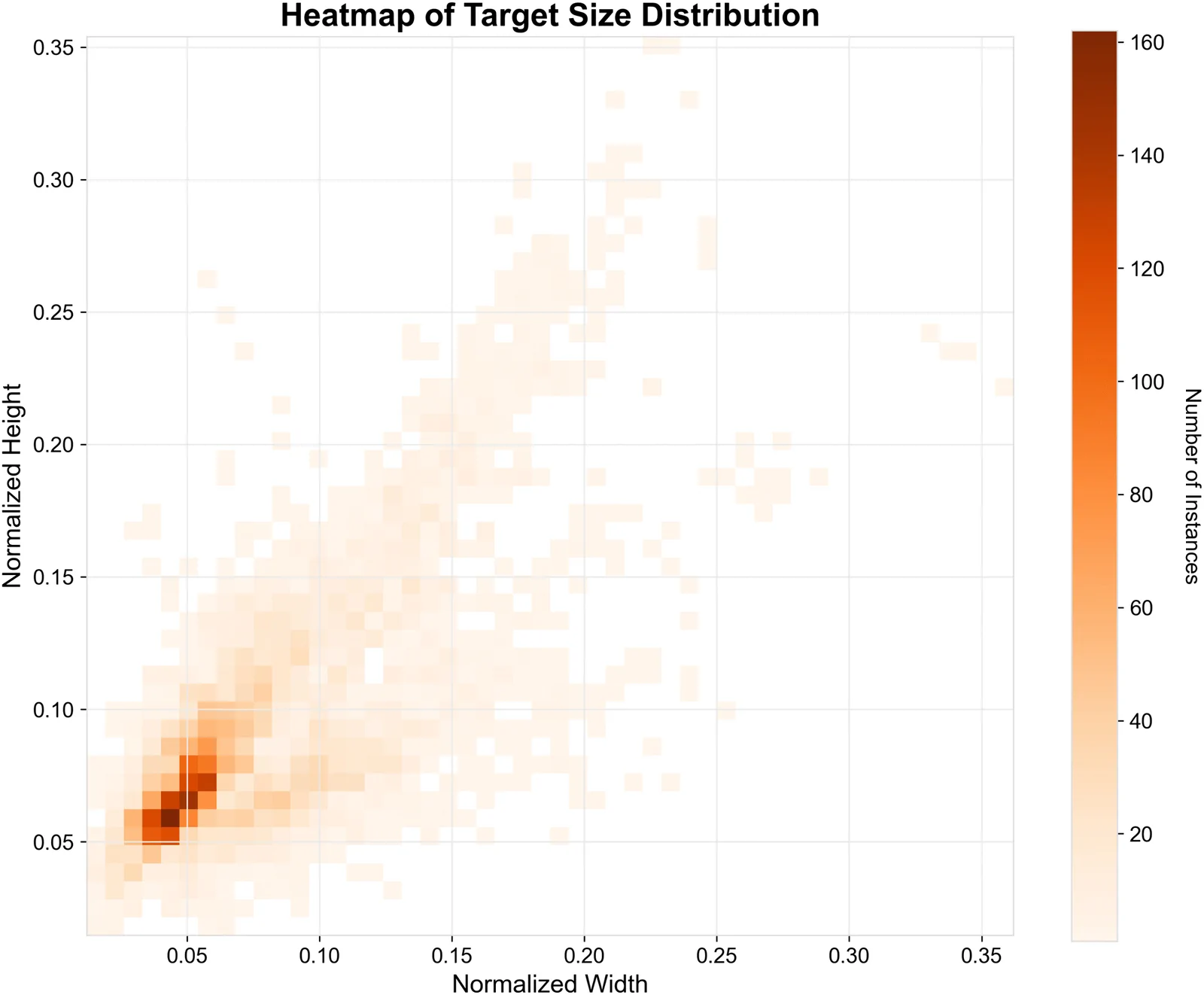
Distribution of normalized bounding box dimensions in the dataset. This highlights the prevalence of small-scale targets.

The specific distribution of annotated instances across the five phenological stages and dataset partitions is detailed in Table 1. This distribution naturally reflects the biological growth cycle of the ‘Juice Balcony’ variety, demonstrating a higher prevalence of mid-stage blooming (Degrees 1 and 2). Furthermore, an analysis of the normalized bounding box dimensions, as shown in Fig 2, highlights the prevalence of small-scale targets, with over 60% of instances occupying less than 10% of the total image area. This confirms the dataset’s validity for evaluating extreme small-target detection capabilities.

**Table 1:** Exact distribution of annotated rose instances across phenological stages.

Phenological Stage	Training Set	Validation Set	In-Domain Test	Out-of-Domain Test
Degree 0	593	185	181	155
Degree 1	1428	447	425	362
Degree 2	1642	513	482	410
Degree 3	779	244	234	193
Degree 4	821	259	241	207
Total Instances	5263	1648	1563	1327

Degrees 0–4 represent the sequential blooming stages of the ‘Juice Balcony’ rose variety, ranging from dormant buds to full senescence. The In-Domain and Out-of-Domain test sets are used to evaluate the model’s generalization across consistent and varying greenhouse environmental conditions, respectively.

#### 2.1.3. Daset partitioning and evaluation protocol.

The 1,002 images from the primary dataset were randomly partitioned into a training set (602 images), a validation set (200 images), and an in-domain test set (200 images), strictly adhering to a 6:2:2 ratio. The 100 images from the domain shift dataset were kept entirely isolated and utilized exclusively for out-of-domain evaluation.

To ensure statistical stability and mitigate partition variance, a 5-fold cross-validation protocol was implemented on the baseline dataset. This strategy validates that the reported model performance reflects the intrinsic generalization capability of the Flora-YOLO architecture, rather than a stochastic artifact of a specific data split. All validation and test sets were maintained in their original, unaugmented state to faithfully mirror real-world operational environments.

### 2.2. The Flora-YOLO model

**Fig 3:**
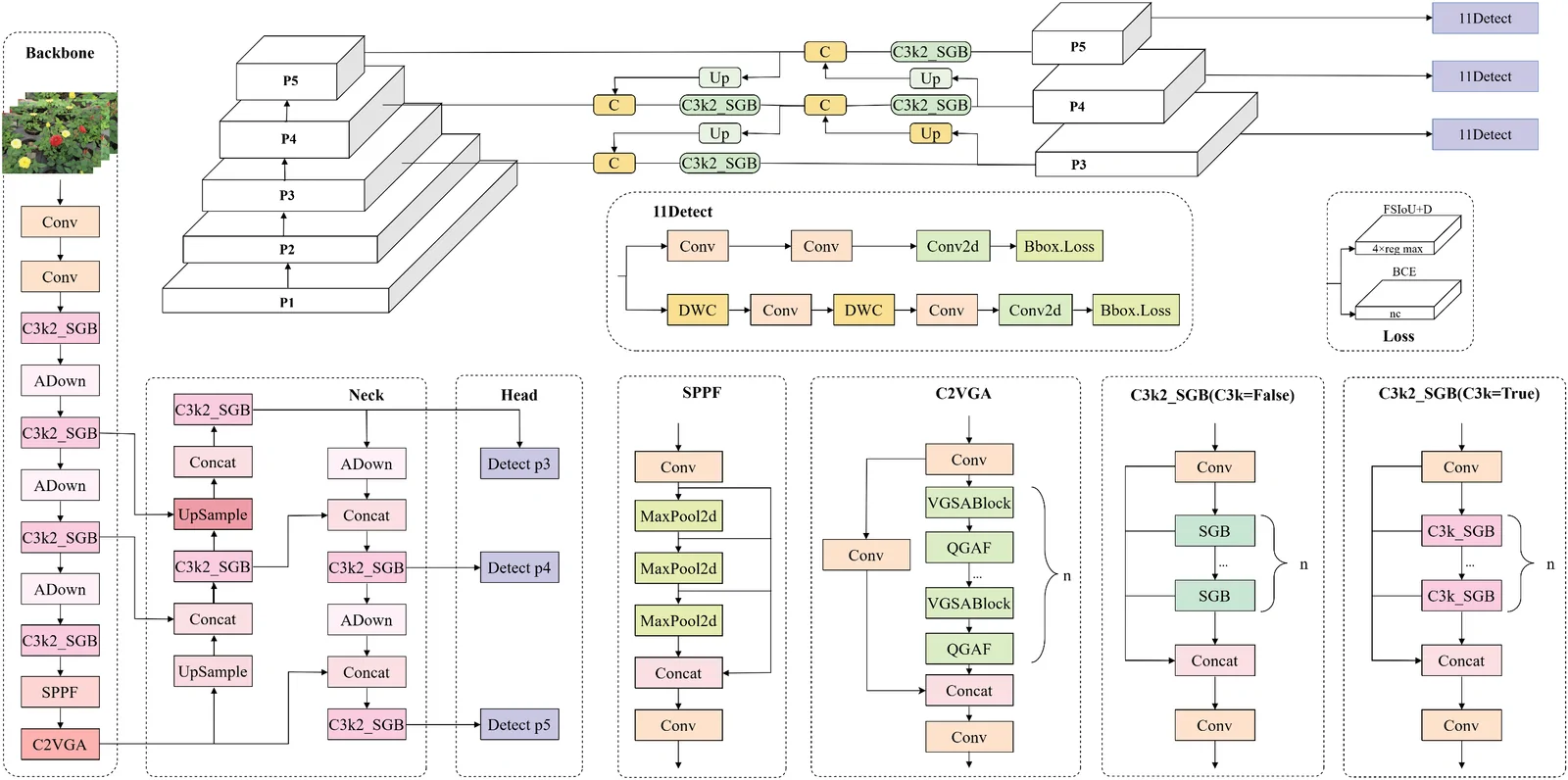
Overview of the proposed Flora-YOLO architecture based on the YOLO11n pipeline. The architecture highlights four key optimizations: the C3k2-based Sandglass-Gated Bottleneck (C3k2_SGB) module in the backbone for fine-grained feature extraction, the ADown (Adaptive Downsampling) module for edge-preserving downsampling, the Cross Stage Partial Variance-Gated and Quadrant-Attention (C2VGA) hybrid attention in the neck for noise suppression, and the Focused Shape-IoU (FSIoU) loss function in the head for shape-aware regression.

Flora-YOLO is a tailored, lightweight detection framework engineered for the fine-grained phenological grading of flowers in complex agricultural environments. Built upon the YOLO11n baseline, the architecture incorporates targeted structural optimizations across its feature extraction, spatial downsampling, semantic fusion, and regression stages. The unified framework, illustrated in Fig 3, is designed to systematically resolve the four primary bottlenecks of agricultural vision while preserving a minimal computational footprint suitable for edge deployment.

The Flora-YOLO framework addresses these manifold agricultural challenges through end-to-end architectural refinements:

To address fine-grained morphological ambiguity (Bottleneck 1), the backbone incorporates the C3k2_SGB module. Through its unique inverted sandglass structure, this module effectively reduces parameter redundancy while strengthening the extraction of subtle, intra-class visual cues—such as delicate petal textures and stamen morphologies—that are essential for distinguishing between adjacent blooming stages.

To mitigate the impact of severe physical occlusion (Bottleneck 2), we integrate the Adaptive Downsampling (ADown) module—originally proposed in the YOLOv9 architecture—into the network’s spatial compression pathway [37]. Unlike standard pooling or strided convolutions that often permanently discard fragmented visual data, ADown utilizes a dual-branch parallel structure. This design adaptively aggregates multi-scale features, effectively preserving critical high-frequency spatial contours (e.g., the visible edges of bisected flowers) during feature map compression.

During the semantic fusion phase in the network’s neck, the C2VGA hybrid attention mechanism is introduced to counter the extreme small-target constraint (Bottleneck 3). By evaluating channel variance to measure information density, this module explicitly amplifies the weak semantic signals of minute, early-stage buds. Concurrently, it suppresses overwhelming background noise, preventing the localized features of small targets from being diluted by complex greenhouse environments.

Ultimately, to overcome the challenges of irregular morphologies and domain shifts (Bottleneck 4), the detection head employs the FSIoU loss function. By incorporating a shape-aware penalty term and an inner-focusing dynamic, FSIoU provides precise gradient guidance for bounding-box regression. This mechanism forces the network to learn boundaries that tightly conform to the true, non-rigid contours of the flowers, significantly improving both convergence stability and final localization precision under varying camera perspectives.

#### 2.2.1. C3k2_SGB feature extraction module.

To resolve the fine-grained morphological ambiguity inherent in rose phenology (Bottleneck 1), as a domain-specific adaptation, we designed the C3k2_SGB (C3k2-based Sandglass-Gated Bottleneck) module by adapting the sandglass-gated concept into the YOLO C3k2 bottleneck structure.

**Fig 4:**
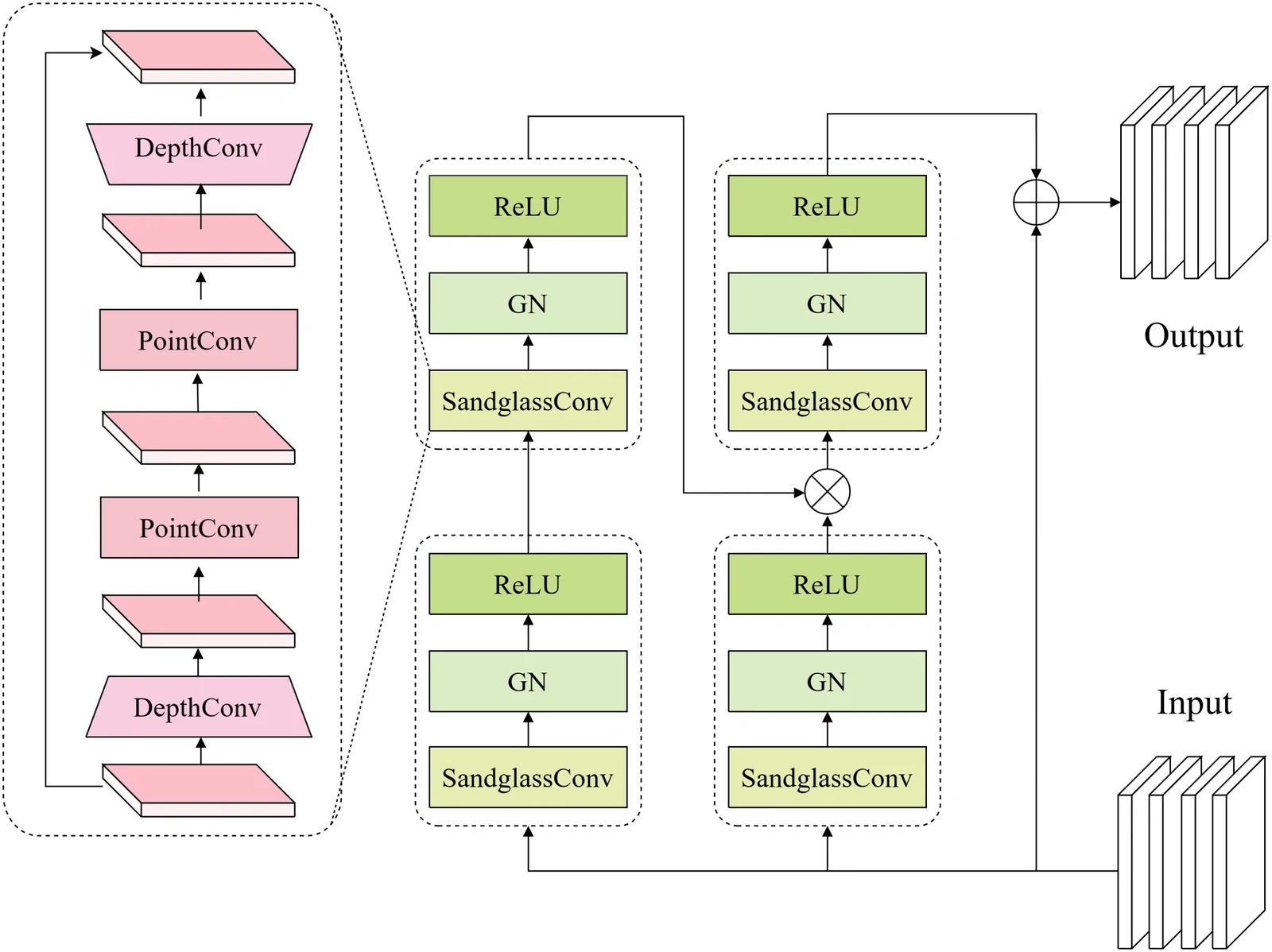
Schematic illustration of the proposed Sandglass-Gated Bottleneck (SGB) module. The left panel details the internal Sandglass Convolution Unit, which utilizes an inverted “high-dim - > low-dim - > high-dim” design to preserve spatial information. The right panel presents the overall architecture, where a dual-path gating mechanism (Main Branch and Gate Branch) is employed to adaptively highlight target features and suppress background noise via element-wise multiplication.

The core of this module is the Sandglass-Gated Bottleneck (SGB), shown in Fig 4. Its design is driven by the need to retain high-resolution spatial details, which are typically lost during the aggressive spatial compression seen in standard lightweight networks.

The pivotal innovation of the SGB module lies in its internal Sandglass Bottleneck Convolution unit. Unlike the traditional bottleneck structure—which follows a “wide-narrow-wide” pattern—we adopted an inverted sandglass structure, specifically a “high-dim (DWConv) - > low-dim (PWConv) - > high-dim (DWConv)” design. Specifically, the input feature map first undergoes a depthwise convolution (DWConv) without changing the channel count, allowing it to fully capture spatial features like floral contours and textures in a higher-dimensional space. Subsequently, a pointwise convolution is used for dimension reduction to compress channel information. In this low-dimensional space, another pointwise convolution performs dimension expansion, restoring channel dimensionality and fusing features. Finally, a second DWConv refines the high-dimensional features, complemented by a residual connection to facilitate information flow and gradient stability. The module structure is formulated as:


$$\begin{array}{c} {\text{X}}_{\text{out}}=\text{Depthwis}{\text{e}}_{\text{2}}(\text{Pointwis}{\text{e}}_{\text{2}}(\text{Pointwis}{\text{e}}_{\text{1}}(\text{Depthwis}{\text{e}}_{\text{1}}\left(\text{x})))\right) \end{array}$$ (1)

The entire SGB module adopts the gating mechanism design from SCSegamba [38]. The input feature x is split into two paths: the main branch (proj and proj2) is responsible for deep feature extraction through two levels of sandglass bottleneck convolution units, generating the feature map $x_{\text{1}}$; the gate branch (proj3) learns a weight mask ${\text{x}}_{\text{2}}$ via a lightweight sandglass bottleneck unit. By performing element-wise multiplication $\left(\text{x}={\text{x}}_{\text{1}}\times {\text{x}}_{\text{2}}\right)$, the gate branch can dynamically and adaptively adjust the features output by the main branch, enhancing focus on the flower target regions while suppressing irrelevant information such as the background. Finally, after refinement by another sandglass bottleneck convolution (proj4) and a residual connection with the original input, the module outputs the final enhanced feature map. Unlike the original SCSegamba, which was engineered for heavy 3D/2D medical image segmentation and relies on high-overhead dense convolutions, our proposed SGB adapts its gating mechanism into parameter-efficient 1D depthwise operations to maintain extremely low computational complexity for edge deployment. This structural transformation is particularly beneficial for automated rose grading under complex greenhouse environments. Standard dense gating operations often induce excessive memory traffic and parameter redundancy on resource-constrained mobile hardware. By decoupling spatial and channel feature filtering into 1D depthwise sandglass bottlenecks, SGB substantially reduces parameters and floating-point operations while preserving the capacity for dynamic feature re-weighting. Consequently, the network effectively filters out persistent background noise—such as dense foliage, shadowing, and structural greenhouse supports—and selectively amplifies minute, fine-grained floral edge textures without compromising real-time inference speed on edge devices.

**Fig 5:**
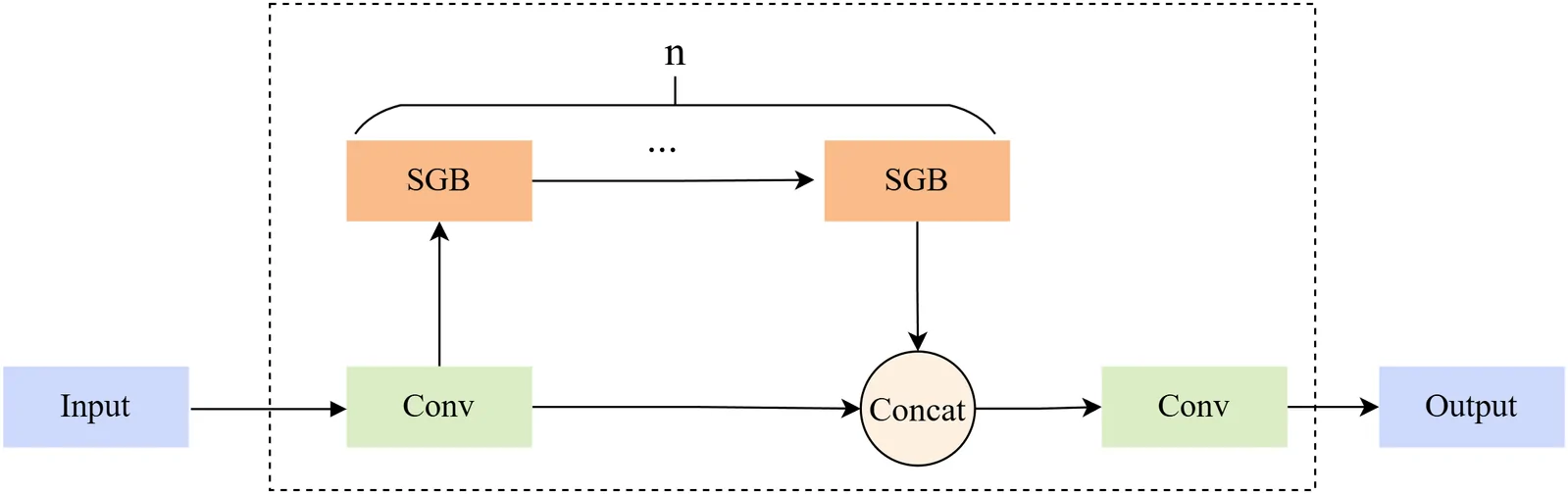
Schematic diagram of the C3k2_SGB module. The architecture integrates the proposed Sandglass-Gated Bottleneck (SGB) units into the C3k2 framework by replacing the original bottleneck components, while preserving the multi-branch design with feature concatenation.

Based on this, we proposed the C3k2_SGB module. In the new C3k2_SGB module, both the Gated_Bottleneck_Convolution unit originally used by the C3k2 module (i.e., the unit used when c3k=False) and the Gated_Bottleneck_Convolution unit within the C3k module it depends on (used when c3k=True) are replaced with our designed Sandglass-Gated Bottleneck (SGB) module, as depicted in Fig 5.

The integration of SGB units into the C3k2 framework creates an optimal balance between computational efficiency and feature discrimination. Structurally, the parameter-efficient characteristics of the SGB module are multiplied through repeated use in the deeper backbone layers. This substantially reduces the total system load, ensuring high-speed inference on resource-limited agricultural edge platforms. Functionally, the synergy between the multi-scale feature extraction of C3k2 and the SGB unit’s ability to capture granular textures—such as stamen shapes and petal curves—effectively mitigates the fine-grained classification challenge. By supplying high-fidelity feature maps to the detection head, the C3k2_SGB module provides stable visual cues, significantly improving the grading precision for morphologically similar blooming stages.

#### 2.2.2. ADown downsampling module.

To prevent the data loss associated with standard spatial scaling—especially the loss of fine edge textures for occluded blooms (Bottleneck 2)—as a structural integration, we integrated the Adaptive Downsampling (ADown) module into the Flora-YOLO network. First proposed in the YOLOv9 architecture, ADown is leveraged here for its strong ability to preserve multi-level features during spatial reduction [33]. Unlike standard pooling or strided convolutions that often discard fragmented pixel data, ADown uses a dual-path design to maintain feature integrity through data fusion.

**Fig 6:**
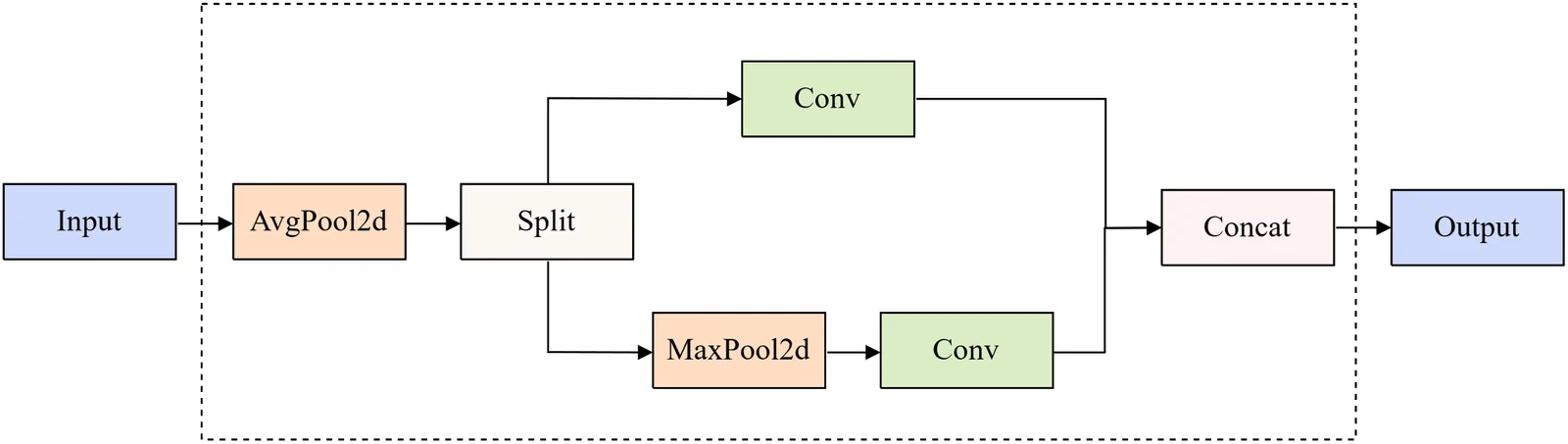
Operational flow of the ADown adaptive downsampling unit. Instead of standard pooling, this design utilizes concurrent processing streams combining average and max pooling to minimize the loss of fine-grained details during spatial reduction.

The operational flow of the ADown unit, shown in Fig 6, starts with an initial global scaling via average pooling to balance the feature distribution. Next, the feature map splits along the channel axis into two parallel paths. The first path captures local salient features by combining max pooling with convolutional layers; this highlights strong structural cues, such as sharp petal edges and stamens, which are vital for tight bounding boxes. Concurrently, the second path retains regional traits through a direct convolutional sequence, preserving smoother, low-level data such as color shifts and diffuse textures that define the bloom stage. The final output is formed by merging these two paths, yielding a feature map that blends sharp structural details with continuous semantic traits.

For the flower grading task, the core benefit of the ADown module is its capacity to preserve “edge-sensitive” data. When floral targets are partly hidden by leaves or densely clustered, these preserved edge contours provide the detection head with the spatial cues needed for precise localization. By utilizing this adaptive feature fusion without introducing a heavy computational load, the ADown unit offers robust support for highly precise grading under the complex conditions of modern agricultural environments.

#### 2.2.3. C2VGA hybrid attention module.

To address the limitations of extreme small targets and severe background noise (Bottleneck 3), as a newly proposed novel contribution, we designed a lightweight hybrid attention structure named C2VGA (CSP-based Variance-Gated Attention). This module replaces the native C2PSA (Cross Stage Partial PSA) in the YOLO11n neck to form a more robust, context-aware feature pyramid. The C2VGA unit integrates two specific attention mechanisms—the VGSABlock (Variance-Gated Self-Attention) and the QGAF (Quadrant-Gated Attention Fusion) module—arranged in an alternating layout within the efficient CSPNet paradigm.In this configuration, the VGSABlock acts as a texture-sensitive filter to amplify weak signals from minute objects by dynamically adjusting feature channel weights. Subsequently, the QGAF module contextualizes these details by fusing global information with multi-scale local features, ensuring a more comprehensive representation of small-scale targets against complex backgrounds.

**Fig 7:**
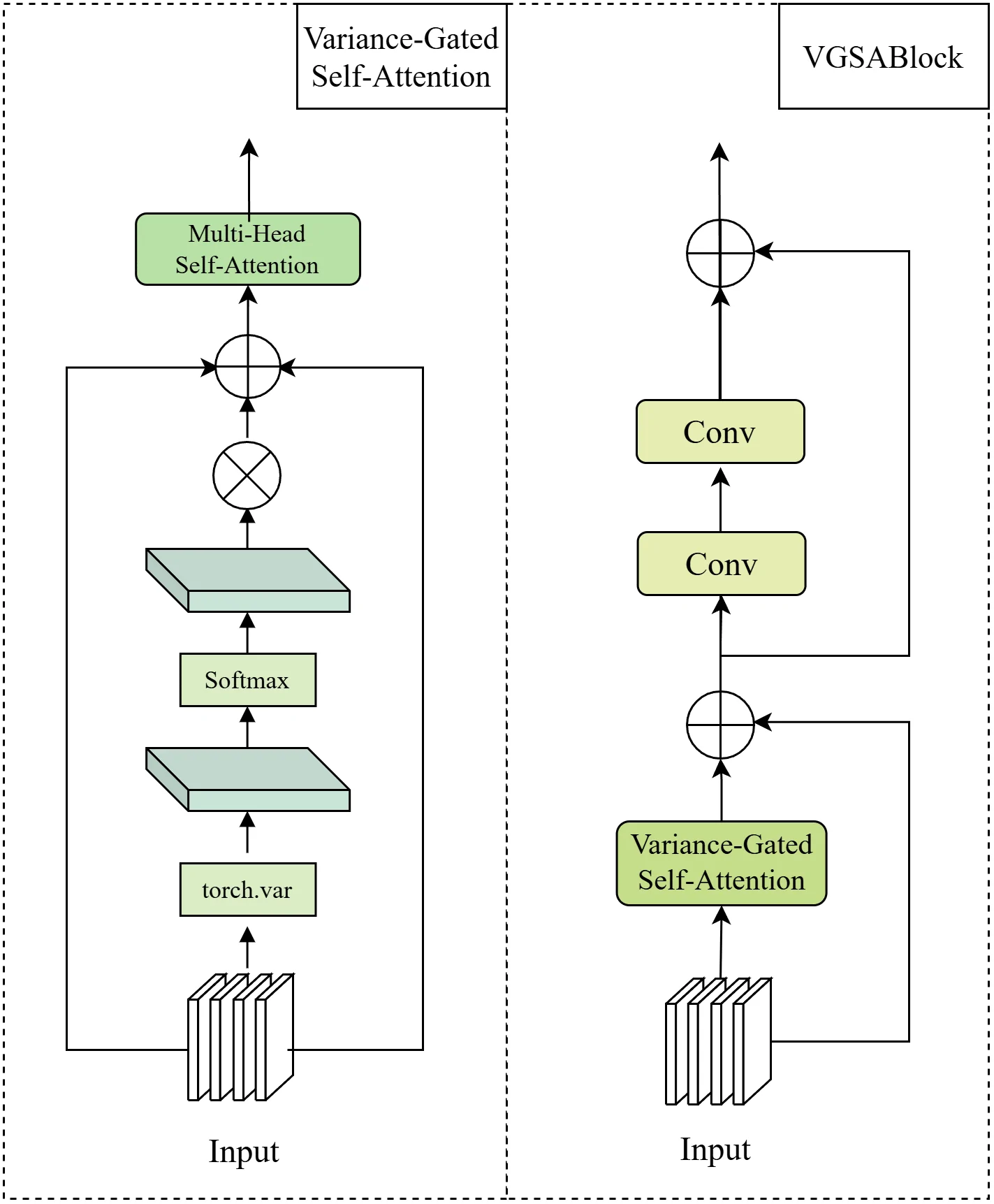
Schematic of the Variance-Gated Self-Attention framework. The left panel illustrates the core attention mechanism employing variance-based channel re-weighting. The right panel presents the complete VGSABlock architecture, which encapsulates the attention unit within a residual structure to serve as the fundamental building block of the C2VGA module.

The VGSABlock (Variance-Gated Self-Attention), whose structure is detailed in Fig 7 is designed to solve a problem in fine-grained tasks like flower recognition: standard self-attention mechanisms treat all feature channels equally, which can lead to computational resources being squandered on low-information regions, such as background noise. Its core principle is that channels with higher variance typically correspond to richer visual details. VGSABlock leverages this through a lightweight, two-stage process. First, the variance-gating mechanism: for an input feature map x, we first calculate the variance $\sigma_{\text{c}}^{\text{2}}$ for each channel c. This is a computationally minimal operation that effectively measures the information density of each channel. Subsequently, we use a Softmax function to normalize these variance scores, generating competitive attention weights w:


$$\begin{array}{c} \text{w}=\text{Soft}\text{max}(\sigma_{\text{1}}^{\text{2}},\sigma_{\text{2}}^{\text{2}},\ldots ,\sigma_{\text{c}}^{\text{2}}) \end{array}$$ (2)

This weight preferentially highlights channels that capture the most unique floral features. The resulting channel-attended feature map is then fed into a multi-head self-attention (MHSA) unit to capture finer spatial dependencies. To maintain the real-time performance of YOLO11n, we introduce lightweight depthwise separable convolutions to generate positional encodings, providing the model with critical positional awareness without significantly increasing computational overhead. Finally, the entire VGSABlock module is encapsulated with a feed-forward network (FFN) and residual connections, forming a complete component that can be integrated into the YOLO11n architecture.

The QGAF Module (Quadrant-Gated Attention Fusion) is used to address the significant scale variations that flower targets exhibit due to different viewpoints and growth stages, as well as the challenges posed by texture similarity and mutual occlusion between petals and the background. Inspired by the LPA module in SwinPA-Net [39], we recognized the effectiveness of processing local and global features in parallel. However, while SwinPA-Net relies on heavy shifted-window self-attention with quadratic memory scaling and simple summation fusion, its complexity limits deployment and risks feature dilution. To resolve these critical limitations, we propose the QGAF module, which combines a zero-parameter channel-variance gating strategy with a multi-kernel quadrant-splitting spatial branch to dynamically fuse multi-scale features without imposing excessive NPU memory burdens. Specifically, SwinPA-Net’s high computational memory footprint and rigid addition-based feature aggregation often lead to severe inference latency on edge platforms and lose subtle, fine-grained semantic cues. In contrast, QGAF replaces window-based self-attention with a parameter-free channel-variance path, operating at linear memory complexity. This branch leverages inter-channel feature variance to dynamically calculate attention weights, effectively highlighting subtle color and texture distinctions between early-stage buds and surrounding foliage. Concurrently, the multi-kernel quadrant-splitting spatial branch captures non-uniform receptive fields across spatial quadrants, allowing the network to handle extreme scale variations caused by shifting camera distances and non-rigid floral expansion. By adaptively gating and weighting these parallel branches before dynamic fusion, QGAF achieves fine-grained multi-scale representation while eliminating memory bottlenecks during hardware inference on resource-constrained NPU accelerators.

**Fig 8:**
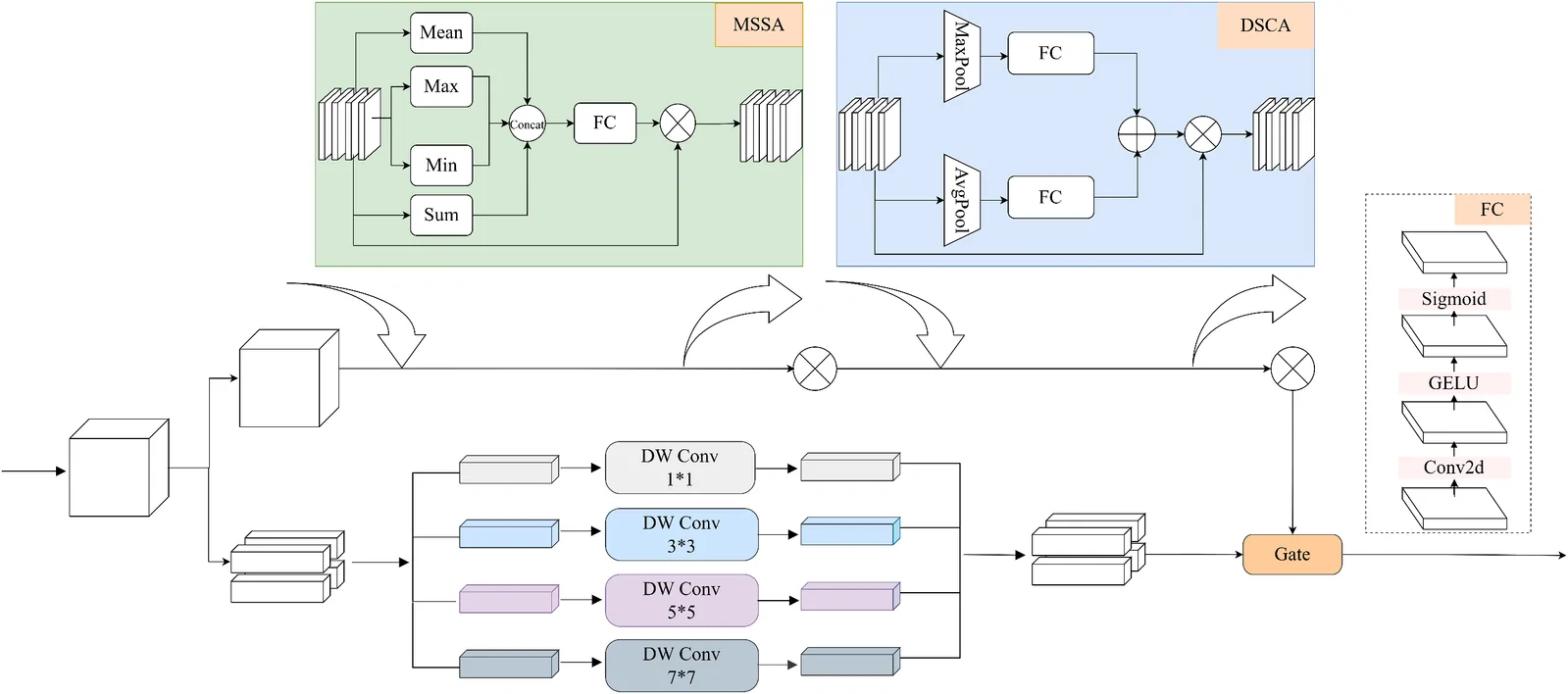
The detailed architecture of the proposed Quadrant-Gated Attention Fusion (QGAF) module. The framework consists of a Global Context Branch (top) for holistic dependency modeling and a Multi-Scale Local Branch (bottom) for extracting fine-grained details via quadrant splitting. A gating mechanism dynamically fuses these outputs to balance global and local information.

The Global Context Branch, as depicted in the top panel of Fig 8, aims to capture the holistic information of the feature map. It consists of our improved DSCA (Dual-Stream Channel Attention) module and MSSA (Multi-Statistical Spatial Attention) module in series. DSCA utilizes a dual-stream design (average and max pooling) for a more comprehensive evaluation of channel weights, while MSSA further fuses four statistical features at the channel dimension (mean, max, min, and sum) to generate a more precise spatial attention map, thereby highlighting key regions.

The Multi-Scale Local Branch is responsible for extracting diverse local details. It first spatially divides the feature map into four quadrants. Then, it applies depthwise separable convolutions with diverse receptive fields (using kernel sizes of 1x1, 3x3, 5x5, and 7x7) to each quadrant in parallel. This spatial-splitting strategy ensures that the model captures multi-scale local patterns across different regions without the prohibitive computational cost of applying all kernels to the entire feature map. The processed quadrant features are then recombined to form a feature map rich in local information, denoted as$\;{\text{x}}_{\text{local}}$.

The core of this module lies in its gated fusion mechanism, which adaptively balances global and local information. The output from the global branch, ${\text{x}}_{global}$, and the output from the local branch, ${\text{x}}_{\text{local}}\;$, are concatenated and fed into a lightweight gating network composed of a 1x1 convolution and a Sigmoid activation function. This generates the spatial gating weights, G. The final output of the module, Y, is calculated as follows:


$$\begin{array}{c} \text{Y}={\text{x}}_{\text{global}}⊗\text{G}+{\text{x}}_{\text{local}}⊗(\text{1}-\text{G}) \end{array}$$ (3)

where ⊗ represents element-wise multiplication.

Finally, the processed attention branch and the bypass branch are concatenated, and a 1 × 1 convolution is used for information fusion to generate the final output of the C2VGA module. Ultimately, through its synergistic process of local feature amplification and global contextualization, the C2VGA module achieves an optimal balance between performance and efficiency. This mechanism significantly enhances the model’s accuracy in detecting and recognizing multi-scale floral targets, particularly extreme small-scale buds, in complex agricultural environments.

#### 2.2.4. FSIoU loss function.

To mitigate the impact of irregular morphologies and domain shifts (Bottleneck 4), as a novel loss function developed in this work, we formulated the Focused Shape-IoU (FSIoU) loss function. In dynamic agricultural environments, varying camera angles and changing lighting conditions heavily distort the perceived 2D shapes of non-rigid blooms. Standard Intersection over Union (IoU) losses focus mainly on box overlap but often overlook shape mismatches, causing loose bounding boxes for floral targets. FSIoU provides shape-aware gradient guidance to enhance bounding-box precision across shifting views.

**Fig 9:**
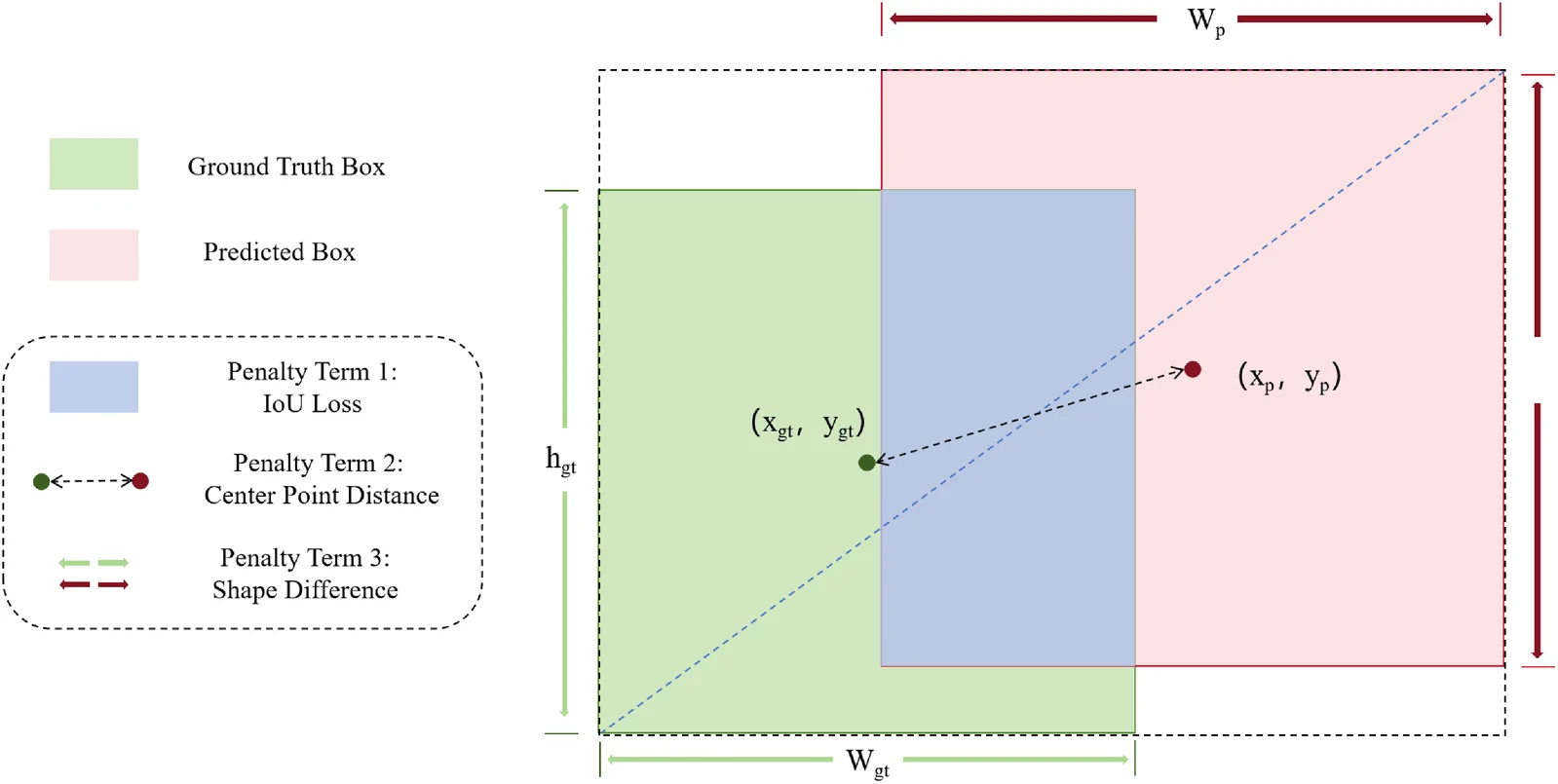
Geometric schematic of the FSIoU loss function components. The diagram illustrates the spatial relationship between the Predicted Box (red) and the Ground Truth Box (green). It visualizes the three core penalty terms integrated into the loss function: the Intersection over Union (IoU) overlap area (blue), the Euclidean distance between center points, and the shape discrepancies based on width and height variations.

The FSIoU loss is designed as a hybrid function that improves upon existing methods by integrating two key concepts: a shape-aware penalty term derived from Shape-IoU [40] and a dynamic focusing mechanism from WIoU [41]. While static Shape-IoU struggles under non-rigid blooming deformations and WIoU targets primarily rigid objects, FSIoU unifies their strengths by incorporating dynamic Inner-IoU auxiliary ratios into shape-aware regression. Specifically, traditional IoU loss functions often experience gradient vanishing or instability when fitting non-rigid, irregular boundaries typical of blooming roses, as aspect ratio penalties alone cannot accommodate non-uniform petal expansion. Furthermore, static WIoU focus mechanisms apply uniform weighting across bounding box regions, making them sensitive to noisy boundary pixels caused by foliage occlusions. To resolve these limitations, FSIoU incorporates a shape-aware component that introduces explicit penalties for discrepancies in center point distance, scale, and aspect ratio, while dynamically weighting regression gradients based on target difficulty. Concurrently, to cope with complex backgrounds and dense flower arrangements, FSIoU utilizes an “Inner-IoU” auxiliary ratio to optimize its detection performance. This mechanism calculates the IoU within an adjustable central sub-region of the bounding box, steering the regression process toward the most semantically representative core of the floral target. By diminishing the influence of ambiguous boundary pixels (e.g., overlapping petal edges, leaf margins, or background shadows), FSIoU prevents bounding-box drift and significantly enhances localization stability under changing camera angles and dense greenhouse crowding. The geometric schematic of the proposed FSIoU loss components is illustrated in Fig 9.

Applying the FSIoU loss to the YOLO11n model significantly enhances its performance on the flower recognition task. The shape-aware penalty term guides the model to learn more precise representations of various flower shapes, thus improving localization accuracy for non-rigid floral structures. Simultaneously, the dynamic focusing mechanism intelligently redistributes the gradient gain based on the “outlierness” of the samples. Instead of blindly increasing weights for all low-IoU samples, this mechanism compels the model to prioritize learning from representative hard samples (such as tiny or partially occluded flowers) while mitigating the negative impact of extreme outliers (e.g., severely noisy or misannotated targets). This balanced focus effectively reduces the missed detection rate and enhances the overall reliability and convergence stability of the recognition system.

The formula for the FSIoU loss function is as follows:


$$\begin{array}{c} {\text{L}}_{\text{FSIoU}}=\text{R}\cdot {\text{L}}_{\text{Shape}-\text{IoU}} \end{array}$$ (4)

Where R is a detachable focusing factor calculated based on IoU, and$L_{Shape-IoU}$represents the shape-aware part of the loss. When Inner-IoU is enabled, the IoU terms in the above formula and in factorRare replaced by$\text{Io}{\text{U}}_{\text{inner}}$. The calculation for $\text{Io}{\text{U}}_{\text{inner}}$ is:


$$\begin{array}{c} \text{Io}{\text{U}}_{\text{inner}}=\frac{{\text{B}}_{\text{inner}}^{\text{p}}\cap {\text{B}}_{\text{inner}}^{\text{gt}}}{{\text{B}}_{\text{inner}}^{\text{p}}\cup {\text{B}}_{\text{inner}}^{\text{gt}}} \end{array}$$ (5)

where${\;\text{B}}_{\text{inner}}\;$is the inner box obtained by scaling the width and height of the original bounding box B=(x,y,w,h) by a specific ratioγ, i.e., ${\text{B}}_{\text{inner}}=(\text{x},\text{y},\text{w}\cdot \gamma ,\text{h}\cdot \gamma )$.

The Shape-IoU loss is defined as:


$$\begin{array}{c} {\text{L}}_{\text{Shape}-\text{IoU}}=\text{1}-\text{IoU}*+\text{dis}\text{tan}\text{c}{\text{e}}_{\text{shape}}+\text{0}.\text{5}\times \Omega_{\text{shape}} \end{array}$$ (6)

where$\text{ Io}{\text{U}}^{*}$is configured as either standard IoU or$\text{ Io}{\text{U}}_{\text{inner}}$. The focusing factor R is dynamically calculated based on the “outlierness” from WIoU, serving to reweight the loss for each sample, and it is a non-monotonic function of$\;(\text{1}-\text{Io}{\text{U}}^{*})$.

The components of the shape penalty term are calculated as follows


$$\begin{array}{c} \text{dis}\text{tan}\text{c}{\text{e}}_{\text{shape}}={\text{h}}_{\text{h}}\times \frac{({\text{x}}_{\text{p}}-{\text{x}}_{\text{gt}}{)}^{\text{2}}}{{\text{c}}^{\text{2}}}+{\text{w}}_{\text{w}}\times \frac{({\text{y}}_{\text{p}}-{\text{y}}_{\text{gt}}{)}^{\text{2}}}{{\text{c}}^{\text{2}}} \end{array}$$ (7)

$$\begin{array}{c} \Omega_{\text{shape}}=(\text{1}-{\text{e}}^{-\omega_{\text{w}}}{)}^{\text{4}}+(\text{1}-{\text{e}}^{-{\text{w}}_{\text{h}}}{)}^{\text{4}} \end{array}$$ (8)

where the horizontal and vertical weight coefficients $w_{w}$and${\;h}_{h}$are:


$$\begin{array}{c} {\text{w}}_{\text{w}}=\frac{\text{2}\times {\text{w}}_{\text{gt}}^{\text{scale}}}{{\text{w}}_{\text{gt}}^{\text{scale}}+{\text{h}}_{\text{gt}}^{\text{scale}}} \end{array}$$ (9)

$$\begin{array}{c} {\text{h}}_{\text{h}}=\frac{\text{2}\times {\text{h}}_{\text{gt}}^{\text{scale}}}{{\text{w}}_{\text{gt}}^{\text{scale}}+{\text{h}}_{\text{gt}}^{\text{scale}}} \end{array}$$ (10)

And the shape difference terms${\;\omega }_{w}$and $\omega_{h}$are:


$$\begin{array}{c} \omega_{\text{w}}={\text{h}}_{\text{h}}\times \frac{\left|{\text{w}}_{\text{p}}-{\text{w}}_{\text{gt}}\right|}{\text{max}({\text{w}}_{\text{p}},{\text{w}}_{\text{gt}})} \end{array}$$ (11)

$$\begin{array}{c} \omega_{\text{h}}={\text{w}}_{\text{w}}\times \frac{\left|{\text{h}}_{\text{p}}-{\text{h}}_{\text{gt}}\right|}{\text{max}({\text{h}}_{\text{p}},{\text{h}}_{\text{gt}})} \end{array}$$ (12)

#### 2.2.5. Symbol definitions.

${\text{B}}_{\text{p}}$and${\text{B}}_{\text{gt}}$: Predicted bounding box and Ground Truth (GT) box, correspondingly.${\text{w}}_{\text{w}}$and ${\text{h}}_{\text{h}}$: Horizontal and vertical weight coefficients.$({\text{x}}_{\text{p}},{\text{y}}_{\text{p}})$ and $({\text{x}}_{\text{gt}},{\text{y}}_{\text{gt}})$: Center point coordinates of the predicted and GT bounding boxes. c: Diagonal distance of the minimum enclosing box covering both boxes.w,h: Width and height dimensions; superscripts p and gtdenote predicted and GT.$\omega_{w}$and$\omega_{h}$: Shape differences in width or height.scale: A learnable scale factor to adjust the focus on aspect ratio.

By integrating a shape-aware penalty, an inner-bounding dynamic, and adaptive weights for hard samples, the FSIoU loss provides a robust solution for localizing non-rigid targets. This shape-aware logic effectively counters domain shifts, ensuring the network tightly conforms to true floral contours despite severe perspective changes in real-world deployments.

## 3. experimental design

### 3.1. Model training environment

The experiments in this study were carried out on a Windows 11 operating system, utilizing an NVIDIA GeForce RTX 4070 Ti GPU with 12GB of dedicated VRAM. The software environment was based on Python 3.12.11, combined with the PyTorch 2.8.0 deep learning framework and CUDA 12.6 for model training.

The model was trained for a total of 300 epochs, with an early stopping patience value set to 50 to prevent overfitting. Specific training parameters were as follows: input image size of 640x640, batch size of 8, and 4 data loading workers. The AdamW optimizer was employed with an initial learning rate (lr0) of 0.001, momentum of 0.9, and weight decay of 0.0005. A cosine annealing strategy was used for learning rate scheduling, with a final learning rate (lrf) of 0.01. Various data augmentation techniques were used during training to enhance the model’s generalization ability, detailed in Table 2, with Mosaic augmentation being deactivated for the final 10 epochs.

**Table 2:** Data augmentation configuration details and parameter settings used in the training phase.

Technique	Parameter Setting	Primary Role
Mosaic	Application Probability = 1.0	Simulates partially occluded targets and small-sized target detection scenarios by stitching four images.
MixUp	Application Probability = 0.1	Improves model robustness to occlusion by linearly interpolating two images.
Copy-Paste	Application Probability = 0.1	Efficiently constructs occlusion scenarios by randomly copying and pasting target instances between different images.
Random Scale	Scaling Range = 0.5	Augments the number of small target samples by randomly scaling images within a 50% to 150% range.
Random HSV (Value)	Brightness Jitter = 0.4	Simulates variable lighting conditions by applying up to 40% random jitter in the brightness (Value) channel.

These augmentation techniques are applied to enhance the model’s ability to recognize roses under diverse greenhouse conditions, such as varying illumination and leaf occlusion. As specified in the training protocol, Mosaic augmentation is deactivated during the final 10 epochs to ensure the model converges on stable, non-stichted image features.

### 3.2. Model evaluation metrics

To objectively and comprehensively evaluate the overall performance of the model proposed in this paper, a series of recognized quantitative metrics are selected for consideration, spanning three dimensions: model effectiveness, computational cost, and operational efficiency. The model’s effectiveness is primarily reflected by its detection accuracy. While basic indicators such as precision, recall, and F1-score are considered for fundamental evaluation, this study primarily employs mean Average Precision (mAP) as the core quantitative metric to measure the model’s comprehensive accuracy in localizing and identifying targets. To synthetically evaluate the model’s overall detection capability across all categories, we calculate the arithmetic mean of the AP values for all classes. This paper adopts two key mAP evaluation standards: mAP@0.5, which is the mAP calculated at a fixed Intersection over Union (IoU) threshold of 0.5, primarily measuring the model’s fundamental detection and localization ability; and mAP@0.5:0.95, which is introduced for a more stringent evaluation. This metric calculates the average of mAP values over a range of IoU thresholds from 0.5 to 0.95 (in steps of 0.05), comprehensively reflecting the model’s robustness under different localization accuracy requirements.

The practical deployment feasibility of the model is also a critical consideration. FLOPs (Floating Point Operations) are used to quantify the model’s computational complexity; a lower FLOPs value typically signifies a more lightweight model design. Parameters serve as a direct measure of model size, referring to the total sum of all learnable parameters in the model, which determines the required storage space. To assess real-time performance on edge devices, Frames Per Second (FPS) is employed to measure inference speed. This metric is crucial for validating the model’s viability in synchronized industrial sorting scenarios.

## 4. Experimental results and analysis

### 4.1. Ablation study

#### 4.1.1. Independent contribution of proposed modules.

To assess the distinct impact of each structural upgrade, we conducted a decoupled ablation study. In this test, each proposed unit—the C3k2_SGB block, ADown module, C2VGA attention, and FSIoU loss—was integrated individually into the native YOLO11n baseline. This setup isolates the exact accuracy gains and computational shifts associated with each specific change, as shown in Table 3.

**Table 3:** Independent contribution of each proposed module on the pure YOLO11n baseline.

Model	mAP@.5 (%)↑	mAP@.5:.95 (%)↑	Params(M)↓	GFLOPs↓
YOLO11n (baseline)	83.3	48.8	2.58	6.3
YOLO11n + C3k2_SGB	83.5	48.9	2.22	5.6
YOLO11n + ADown	84.1	49.3	1.85	5.1
YOLO11n + C2VGA	84.2	49.4	2.58	6.3
YOLO11n+FSIOU	84.6	49.5	2.58	6.3

Every module yields a positive gain over the pure baseline, demonstrating their standalone efficacy. On the precision front, the FSIoU loss yields the most substantial improvement (+1.3% mAP@0.5) by offering shape-aware bounding box regression for non-rigid floral targets (Bottleneck 4). Likewise, the C2VGA module (+0.9% mAP@0.5) acts as a robust replacement for the native C2PSA. By tuning channel weights, it amplifies weak semantic signals from minute targets (Bottleneck 3).

On the efficiency front, the structural upgrades show exceptional lightweight characteristics. The ADown module improves precision (+0.8% mAP@0.5) while reducing the computing load by 1.2 GFLOPs. This confirms its capacity to preserve vital edge contours for occluded blooms (Bottleneck 2). Also, replacing native blocks with the C3k2_SGB setup drops parameters to 2.22 M and computing to 5.6 GFLOPs, confirming its capability in capturing fine floral textures (Bottleneck 1). In sum, this isolated test proves that each unit solves a unique agricultural challenge, laying a solid foundation for the final Flora-YOLO model.

#### 4.1.2. Progressive integration and synergistic effects.

To quantify the joint impact of our proposed upgrades, we conducted a progressive integration study. By sequentially incorporating the four core modules—C3k2_SGB, ADown, C2VGA, and FSIoU—onto the YOLO11n baseline, we evaluated the transition from the native network to the final Flora-YOLO model. This progressive evaluation reveals the exact performance trajectory and the combined value of all components, as detailed in **Table 4**.

**Table 4:** Ablation experiment results for Flora-YOLO modules.

Model	C3k2_SGB	ADown	C2VGA	FSIoU	mAP@.5 (%)↑	mAP@.5:.95 (%)↑	Params(M)↓	GFLOPs↓
YOLO11n (baseline)					83.3	48.8	2.58	6.3
M1	√				83.5	48.9	2.22	5.6
M2	√	√			84.4	49.3	1.73	4.5
M3	√	√	√		85.1	49.8	1.73	4.5
Flora-YOLO (Ours)	√	√	√	√	86.7	50.4	1.73	4.5

Incorporating the C3k2_SGB module in Model M1 initiated the structural optimization, reducing parameters and computing load while improving the baseline mAP via fine-grained texture extraction (Bottleneck 1). Next, adding the ADown module in Model M2 yielded a substantial improvement in model compression. By replacing standard strided convolutions with ADown’s dual-path design, M2 reduced parameters to 1.73 M and computing to 4.5 GFLOPs. This proves ADown not only reduces parameter redundancy but safely retains the sharp edge contours of occluded blooms (Bottleneck 2).

Building upon this lightweight architecture, the C2VGA hybrid attention in Model M3 increased the mAP@0.5 to 85.1%. Designed to replace native attention nodes without introducing additional computational overhead, C2VGA maintains the computing load flat. It pairs with early modules by tuning channel weights to focus the network on extremely small targets (Bottleneck 3) that might otherwise be diluted in deep feature layers.

Finally, the complete Flora-YOLO model, equipped with the FSIoU loss, achieved a peak performance of 86.7% mAP@0.5. Since FSIoU only steers the training phase, the model keeps its fast inference speed. FSIoU complements the upstream feature enhancements by offering shape-aware bounding box regression for non-rigid roses (Bottleneck 4), yielding a total 3.4% precision jump over the baseline.

For real-world agricultural deployments, this joint design offers substantial operational gains. By reducing the computing load by 28.6%, the model enables edge devices to achieve higher frame rates. This efficiency leap also yields lower power draw, saving crucial battery life for edge hardware in humid greenhouse environments. These staged upgrades perfectly balance precision and speed, boosting the practical value of automated floral grading.

#### 4.1.3. Spatial layout and stacking depth analysis of the SGB module.

To systematically determine the optimal deployment of the Sandglass-Gated Bottleneck (SGB) unit, we expanded our ablation study across two architectural dimensions: the internal integration logic within the C3k2 module and the spatial layout across the network (Backbone versus Neck). This empirical analysis demonstrates how the “Global Replacement” strategy serves as the computationally optimal configuration for miniature rose grading, as presented in **Table 5**.

**Table 5:** Performance evaluation of SGB integration strategies and spatial layouts.

Model	Spatial Layout	Internal Strategy	mAP@.5 (%)↑	GFLOPs↓
Strategy 1	Global	SGB in C3k only	85.1	5.0
Strategy 2	Global	SGB in C3k2 only	85.8	4.9
Strategy 3 (Ours)	Global	Full SGB Replacement	86.7	4.5
Strategy 4	Backbone-Only	Full SGB Replacement	86.0	4.8
Strategy 5	Neck-Only	Full SGB Replacement	85.3	4.9

The initial testing phase focused on the internal integration logic (Strategies 1, 2, and 3). The results demonstrate that the uniform “Full SGB Replacement” (Strategy 3) significantly outperforms partial replacement configurations, achieving the highest detection accuracy (86.7%) and the lowest computational cost (4.5 GFLOPs). This confirms that the inverted sandglass structure is most efficient when fully utilized within the C3k2 framework, maximizing the parameter-efficient characteristics of the dual-path gating mechanism.

The second phase evaluated the spatial layout of the SGB modules across the macro-architecture. Restricting the SGB modules exclusively to the Backbone (Strategy 4) yielded an mAP@0.5 of 86.0%. While effective, the slight degradation compared to the global strategy highlights the importance of SGB units during the feature fusion stage. The backbone successfully preserves fine-grained visual cues—such as delicate petal textures (Bottleneck 1)—but without SGB units in the neck, the network exhibits reduced capacity to filter out complex greenhouse background noise during multi-scale semantic fusion.

Conversely, restricting the SGB modules solely to the Neck (Strategy 5) resulted in a more severe performance drop (85.3%). This gap underscores a critical principle in fine-grained detection: high-fidelity feature fusion in the neck is heavily dependent on the quality of initial feature extraction. When standard convolutions are utilized in the backbone, the subtle micro-features of small buds are often prematurely degraded. Consequently, the advanced gating mechanism in the neck receives sub-optimal inputs, unable to recover the critical textures that were already lost during the early stages of the backbone.

Regarding the module’s stacking depth, our architectural search confirmed that adhering to the baseline’s optimal block repetition depth provided the most stable gradient flow. Experimenting with increased stacking depth within the C3k2_SGB framework yielded marginal accuracy fluctuations while consistently introducing disproportionate increases in latency, thereby compromising the stringent real-time deployment constraints required for mobile agricultural platforms. Ultimately, the Global Replacement (Strategy 3) establishes a continuous, high-fidelity feature pipeline, proving to be the optimal configuration for automated floriculture.

#### 4.1.4. comparative analysis and visualization of attention mechanisms.

To quantitatively evaluate the effectiveness of the C2VGA module, we conducted a direct one-to-one replacement experiment on the YOLO11n baseline, as detailed in **Table 6**. Under an identical parameter budget and computational cost, replacing the native C2PSA with the C2VGA module independently improved the mAP@0.5 from 83.3% to 84.2%. This isolated performance gain confirms the intrinsic suitability of C2VGA for this specific detection task.

**Table 6:** Performance comparison of C2VGA versus native C2PSA on the YOLO11n baseline.

Model	Core Module	mAP@.5 (%)↑	mAP@.5:.95 (%)↑
YOLO11n (Baseline)	C2PSA	83.3	48.8
YOLO11n + C2VGA	C2VGA (Ours)	84.2	49.4

Furthermore, to validate the domain-specific adaptability of C2VGA, we replaced the internal attention mechanism within the network’s C2 bottleneck with other mainstream mechanisms, forming C2PSA_CBAM [42], C2PSA_ECA [43], C2PSA_SimAM [44], C2PSA_EMA [45], C2PSA_BiFormer [46], and C2PSA_CPCA [47]. As shown in **Table 7**, conventional mechanisms such as CBAM and ECA resulted in performance degradation (83.9% and 83.7% mAP@0.5, respectively) compared to the M2 baseline. This decline is likely due to their reliance on global average pooling, which tends to over-smooth the delicate, non-rigid contours of miniature roses that are essential for stage classification. Modern mechanisms like EMA and CPCA yielded competitive results; notably, CPCA matched C2VGA at 85.1% mAP@0.5, but C2VGA achieved superior overall localization precision across stricter IoU thresholds (49.8% mAP@.5:.95).

**Table 7:** Performance comparison of C2VGA module with different attention mechanisms.

Model	mAP@.5 (%)↑	mAP@.5:.95 (%)↑
M2 (Baseline)	84.4	49.3
+ C2PSA_CBAM	83.9	48.6
+ C2PSA_ECA	83.7	48.2
+ C2PSA_SimAM	84.6	49.4
+ C2PSA_EMA	84.7	49.5
+ C2PSA_BiFormer	84.5	49.3
+ C2PSA_CPCA	85.1	49.6
+C2VGA (Ours)	85.1	49.8

In contrast, C2VGA achieved the optimal balance of overall detection and bounding-box regression precision (85.1% mAP@0.5 and 49.8% mAP@0.5:.95). Paired t-tests across 5-fold cross-validation runs confirmed that the performance gain of C2VGA over the baseline M2 (+0.7%) and standard C2PSA (+0.9% in overall framework) is statistically significant (p<0.05). Its variance-gated mechanism avoids information dilution by utilizing channel variance to measure local texture complexity, ensuring that higher weights are assigned to channels capturing the most distinctive floral signals while suppressing cluttered background noise (Bottleneck 3).

**Fig 10:**
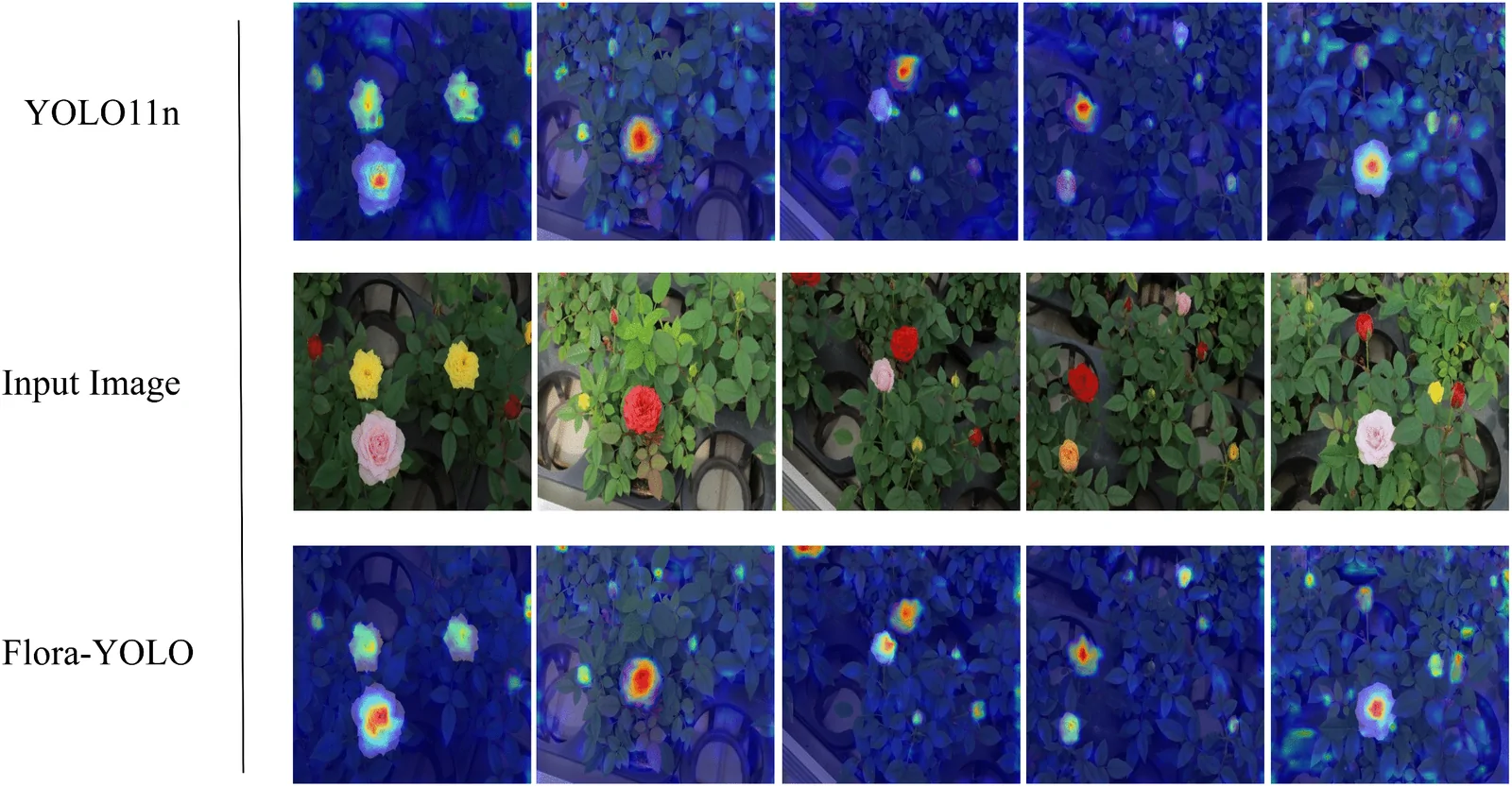
Class activation mapping (CAM) heatmap comparison. The visualization contrasts the diffuse attention of the baseline YOLO11n (top row) with the sharply focused attention of Flora-YOLO (bottom row) based on the same input images (middle row). Flora-YOLO demonstrates superior background noise suppression and tighter bounding around small and occluded floral targets.

To provide a qualitative basis for these performance gains and enhance model interpretability, we employed Class Activation Mapping (CAM) to visualize the feature-focusing behavior. As depicted in Fig 10, the heatmap activations for the baseline YOLO11n (top row) are relatively diffuse, frequently dispersing attention toward non-target background elements such as greenhouse foliage and cultivation trays.

Conversely, Flora-YOLO (bottom row) demonstrates a highly compact and sharply focused activation pattern. Driven by the variance-gating and quadrant-attention mechanisms, the activation regions are strictly concentrated on core discriminative features, such as petal layers and stamens. Even under severe occlusion or when detecting extremely small targets (Bottleneck 3), the model maintains precise focal alignment. This visual evidence confirms that C2VGA effectively suppresses background interference and extracts robust spatial features, enabling highly reliable detection for multi-stage flower grading.

#### 4.1.5. Shape-aware localization analysis of FSIoU.

To evaluate the localization efficacy of the proposed FSIoU loss function, we compared its performance against several state-of-the-art bounding box regression metrics (ShapeIoU, MPDIoU [48], and Wise-IoU) on the M3 baseline.

As presented in Table 8, replacing the baseline CIoU with the FSIoU loss increased the mAP@0.5 to 86.7%, outperforming the strongest competitor, Wise-IoU, by 0.5 percentage points. The structural advantage of FSIoU is particularly evident when contrasted with the performance degradation observed under MPDIoU. As MPDIoU relies on rigid, point-to-point distance penalties, it struggles with fully bloomed roses, which are fundamentally non-rigid targets exhibiting highly variable morphological deformations (Bottleneck 4). Rigid metrics strictly penalize these natural geometric deviations, leading to suboptimal convergence. In contrast, the shape-aware dynamic focusing of FSIoU proves highly adaptable to irregular floral contours.

**Table 8:** Performance comparison of FSIoU with advanced IoU loss functions.

Model	mAP@.5 (%)↑	mAP@.5:.95 (%)↑
M3 (Baseline, CIoU)	85.1	49.8
+ ShapeIoU	85.9	50.0
+ MPDIoU	84.6	49.3
+ Wise-IoU	86.2	50.1
+ FSIoU (Ours)	86.7	50.4

**Fig 11:**
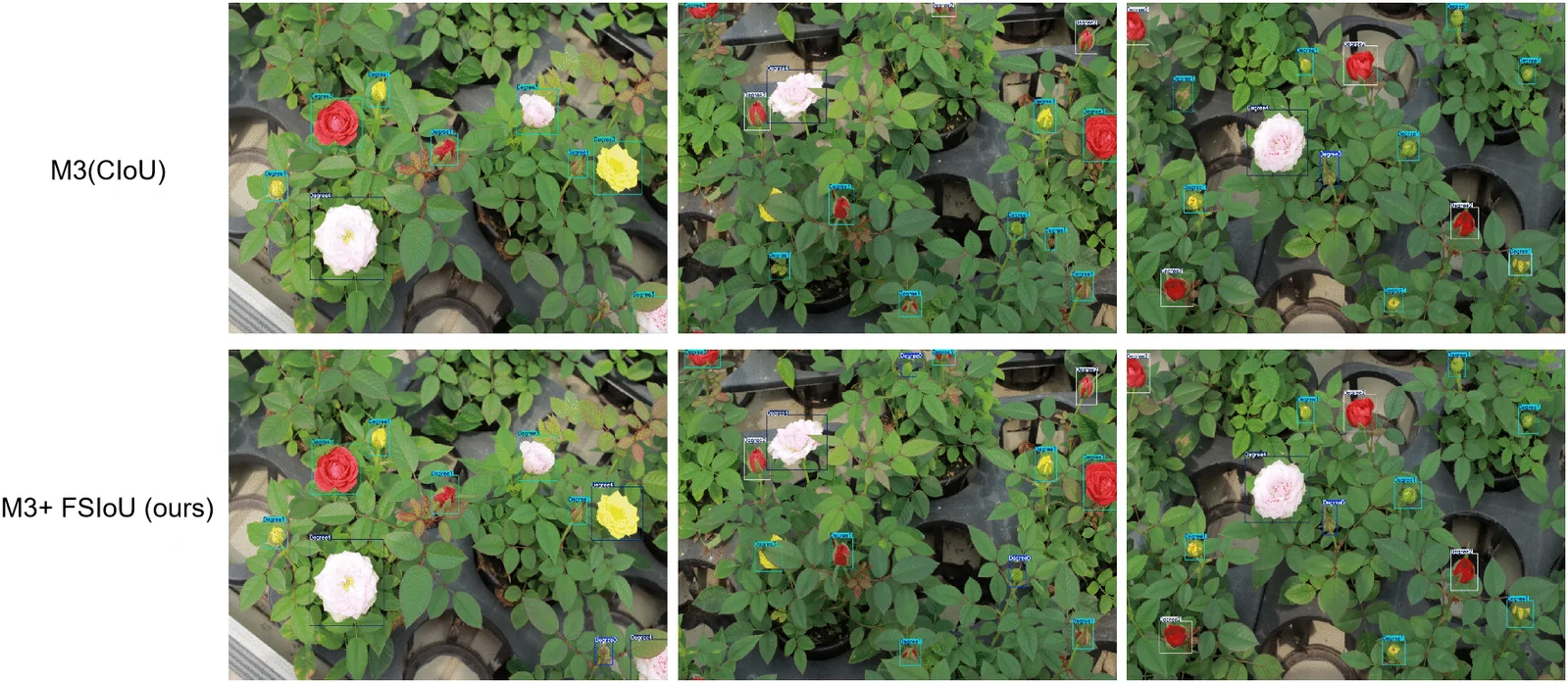
Visual comparison of detection results between the M3 baseline with CIoU (top row) and the final Flora-YOLO with FSIoU (bottom row). The visualization demonstrates FSIoU’s superior capability in tightly delineating non-rigid boundaries, retrieving severely occluded targets, and rejecting morphological false positives caused by background foliage.

Visual inspection, as illustrated in Fig 11, further substantiates this analysis. A critical flaw of the CIoU baseline is its tendency to generate loose bounding boxes around fully bloomed flowers, often incorporating excessive background foliage. This inclusion of noise can induce fine-grained misclassifications, such as confusing adjacent blooming stages. FSIoU’s shape-aware mechanism generates remarkably tight bounding boxes that strictly conform to non-rigid floral boundaries, successfully rectifying classification errors caused by background interference.

Furthermore, FSIoU demonstrates superior robustness under partial occlusion by shifting the regression focus toward inner-similarity and core morphological features. This mechanism allows the model to recover obscured targets that traditional metrics fail to detect while effectively rejecting morphological mimics—background foliage with bud-like aspect ratios—by prioritizing intrinsic shape consistency. Consequently, FSIoU provides robust localization guidance that seamlessly adapts to irregular morphologies and background interference, solidifying the Flora-YOLO architecture for high-precision automated grading in complex greenhouse environments.

### 4.2. Statistical validation and overfitting risk analysis

To ensure the robustness of the Flora-YOLO model and assess the risk of overfitting in fine-grained classification, we conducted a comprehensive statistical validation.

In object detection, model capacity is fundamentally driven by instance-level abundance rather than raw image counts. The baseline dataset provides 8,474 annotated floral instances distributed across five phenological stages. This translates to a dense distribution of localized feature samples within the network’s receptive fields. Such instance-level diversity introduces substantial intra-class variance—encompassing varying scales, partial occlusions, and fluctuating illumination—establishing a robust foundation for feature extraction while mitigating the risk of overfitting.

Furthermore, to validate the statistical stability of the proposed architecture and account for the variance associated with a single data split, a 5-fold cross-validation protocol was executed on the baseline dataset. The dataset was partitioned into five mutually exclusive folds for iterative training and validation. The results are summarized in **Table 9**.

**Table 9:** 5-Fold cross-validation results of Flora-YOLO on the baseline dataset.

Fold	mAP@.5 (%)↑	mAP@.5:.95 (%)↑
Fold 1	86.7	50.4
Fold 2	86.5	50.3
Fold 3	86.8	50.8
Fold 4	86.1	49.9
Fold 5	86.9	50.5
Mean ± SD	86.60 ± 0.32	50.38 ± 0.33

These results demonstrate that Flora-YOLO achieves a mean mAP@0.5 of 86.60% with a low standard deviation of 0.32%. This narrow variance confirms that the model’s high performance derives from the structural stability of its optimized architecture, rather than stochastic variations of a specific data partition.

Finally, an analysis of the convergence dynamics across all five folds reveals no evidence of overfitting. Throughout the training cycles, validation metrics improved synchronously with training metrics, reaching stable plateaus without divergence. This consistent convergence, supported by standard data augmentation strategies (e.g., mosaic augmentation and color jittering), indicates that Flora-YOLO effectively generalizes to novel morphological patterns instead of merely memorizing the training distribution.

### 4.3. Comparison with different object detection models

To evaluate the competitiveness of the Flora-YOLO model for fine-grained flower grading, we conducted a benchmark against representative architectures across various technical paradigms. The selected models are categorized into three tiers: large-scale architectures (to establish theoretical upper bounds), standard generic models (the YOLO series from v3 up to the state-of-the-art YOLO26n), and specialized lightweight variants (including Ghost-YOLO11n [49], Hyper-YOLO-n [50], MS-YOLO [51], and Mamba-YOLO [52]). All evaluations were executed under identical dataset splits and training protocols to ensure a controlled and fair comparison, as demonstrated in **Table 10**.

**Table 10:** Performance and efficiency comparison of Flora-YOLO with mainstream object detection models.

Model	mAP@.5 (%)↑	mAP@.5:.95 (%)↑	Params(M)↓	GFLOPs↓
Faster R-CNN	81.3	47.1	137.10	370.2
RT-DETR-L	86.5	50.1	31.99	103.5
RT-DETR-X	87.2	51.3	65.57	222.5
YOLOv3-tiny	79.7	46.9	12.13	18.9
YOLOv5n	81.9	47.6	2.50	7.1
YOLOv8n	82.7	47.4	2.14	6.0
YOLOv9-tiny	83.1	48.5	1.97	7.6
YOLOv10n	82.5	47.2	2.11	6.5
YOLO11n	83.3	48.8	2.58	6.3
YOLO12n	82.4	48.3	2.34	5.8
YOLO26n	85.5	49.6	2.40	5.4
Ghost-YOLOv11n	83.9	49.1	2.16	5.5
Hyper-YOLO-n	85.8	49.7	4.00	11.4
MS-YOLO	84.2	48.8	2.35	6.7
Mamba-YOLO	85.3	49.5	1.97	4.6
Flora-YOLO(Ours)	86.7	50.4	1.73	4.5

“↑” denotes higher is better; “↓” denotes lower is better. The benchmarked models are categorized into four groups for comprehensive evaluation: (1) Two-Stage Detector (Faster R-CNN); (2) End-to-End Real-Time Transformers (RT-DETR-L/X); (3) Standard Single-Stage YOLO Baselines (YOLOv3-tiny to YOLO26n); and (4) Lightweight/Modern Specialized Architectures (Ghost-YOLOv11n, Hyper-YOLO-n, MS-YOLO, Mamba-YOLO). All models were evaluated under identical training and testing protocols.

Flora-YOLO establishes a significant advantage in balancing detection precision and computational efficiency. Compared to its direct baseline (YOLO11n), Flora-YOLO delivers a substantial improvement of +3.4% in mAP@0.5. More importantly, when benchmarked against the advanced YOLO26n, Flora-YOLO not only achieves superior accuracy (+1.2% mAP@0.5) but also reduces the parameter footprint by 27.9% and the computational load by 16.7%. This demonstrates that architectural optimizations tailored to specific biological traits can outperform the latest general-purpose detectors.

The comparison with specialized lightweight variants further highlights Flora-YOLO’s structural efficiency. While architectures like Hyper-YOLO-n and Mamba-YOLO exhibit robust feature extraction, Flora-YOLO surpasses them with an accuracy of 86.7%. Crucially, Flora-YOLO’s parameter count (1.73 M) and GFLOPs (4.5) are the lowest among all evaluated models. These results indicate that our gated optimizations—which prioritize the preservation of granular floral textures—are more effective for horticultural applications than adopting general lightweight modifications. This establishes a new performance-efficiency Pareto frontier for edge-oriented agricultural vision.

Remarkably, Flora-YOLO exhibits competitive “cross-tier” performance against large-scale paradigms. It surpasses the Transformer-based RT-DETR-L in mAP@0.5 while utilizing merely 5.4% of its parameters. Even against the high-complexity RT-DETR-X, Flora-YOLO maintains a marginal precision gap of only 0.5 percentage points despite being nearly 38 times smaller. This significant reduction in structural complexity without a commensurate loss in accuracy demonstrates that the proposed modules successfully resolve the core agricultural bottlenecks (occlusion, small targets, and fine-grained similarity). Consequently, Flora-YOLO provides a highly viable, real-time solution for large-scale agricultural monitoring on resource-constrained embedded hardware.

**Fig 12:**
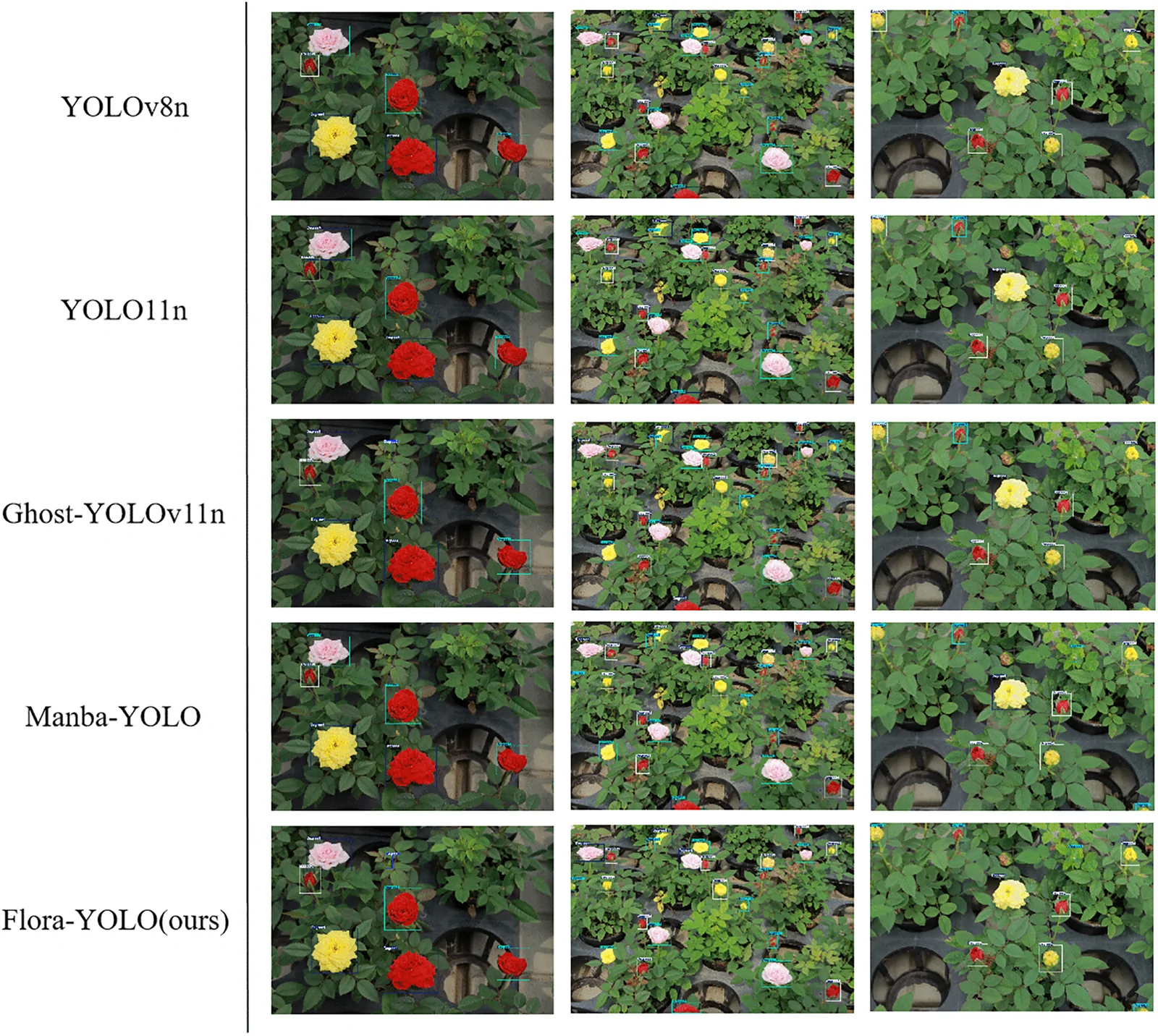
Qualitative comparison of inference results among representative object detection models. The visualizations highlight Flora-YOLO’s enhanced capability in detecting extremely small targets and reducing stage misclassifications in dense, complex greenhouse environments compared to generic baseline models.

To further provide qualitative insights into these overall performance gains, Fig 12 illustrates the inference results of representative models in complex greenhouse scenarios. As visually evident, standard baselines frequently struggle with fine-grained misclassifications of adjacent growth stages and tend to miss extremely small targets (e.g., the minute Degree 0 bud in the first column). In contrast, Flora-YOLO, alongside Mamba-YOLO, demonstrates superior robustness in localizing early-stage buds and maintaining classification accuracy amidst dense foliage. While inherent challenges like severe occlusion still cause occasional missed detections across all models, Flora-YOLO overall exhibits a more precise and stable visual perception, corroborating the quantitative advantages discussed above.

### 4.4. Generalization capability verification

#### 4.4.1. Domain shift robustness with statistical testing.

To evaluate the generalization capacity of the Flora-YOLO framework and verify its robustness in unstructured real-world environments, we conducted a targeted domain shift experiment. Models trained exclusively on the primary benchmark dataset were evaluated directly on a completely unseen out-of-domain dataset without further fine-tuning. This target domain introduces severe environmental variations, including divergent lighting conditions, unfamiliar background infrastructure, and distinct image acquisition devices. To ensure the statistical reliability of the results, we supplemented standard point-estimates with empirical bootstrapping (resampling with replacement) to calculate 95% Confidence Intervals (CI) and establish the significance of performance differences, as detailed in **Table 11**.

**Table 11:** Statistical performance comparison on benchmark and domain-shifted datasets.

Model	Benchmark mAP@.5:.95 (%)↑	Domain-Shifted mAP@.5:.95 (%)↑	95% CI (Shifted)	Δ mAP (%)↓
YOLOv3-tiny	46.9	44.6	[43.8, 45.3]	−2.3
YOLOv5n	47.6	45.8	[44.9, 46.6]	−1.8
YOLOv8n	47.4	45.8	[44.8, 46.5]	−1.6
YOLO11n	48.8	47.0	[46.1, 47.7]	−1.8
Flora-YOLO (Ours)	50.4	49.6	[48.9, 50.0]	−0.8

The domain shift induced performance degradation across all evaluated architectures. However, Flora-YOLO demonstrates superior architectural resilience. Its mAP@0.5:0.95 decreased by only 0.8 percentage points, exhibiting significantly lower sensitivity to domain variations than the baseline models, which suffered drops between 1.6 and 2.3 points. Notably, Flora-YOLO’s performance under severe domain shift (49.6%) still surpasses the in-domain benchmark performance of the native YOLO11n baseline (48.8%).

From a statistical perspective, the bootstrapping analysis confirms that the generalization advantage of Flora-YOLO is not a stochastic artifact. The lower bound of Flora-YOLO’s 95% CI (48.9%) remains strictly higher than the upper bound of the YOLO11n baseline (47.7%), indicating a non-overlapping performance gap. A subsequent paired permutation test yielded a p-value of p<0.01, significantly rejecting the null hypothesis that the models perform equally on out-of-domain data.

This high degree of statistical stability validates the intrinsic robustness of our architectural design. It confirms that the ADown module effectively preserves high-frequency edge information, while the C2VGA mechanism successfully suppresses unfamiliar environmental noise. These integrated features allow the network to extract invariant morphological representations rather than overfitting to source-domain environmental biases. Ultimately, Flora-YOLO offers a statistically sound and reliable solution for diverse real-world agricultural deployments across varying facility types.

#### 4.4.2. Extensive validation on public agricultural datasets.

To evaluate the universality of the Flora-YOLO framework and ensure its architectural advantages are not restricted to a specific dataset, we conducted extensive cross-domain validations on two distinct public agricultural benchmarks. These datasets encompass diverse crop species, varied cultivation environments (greenhouses and orchards), and different phenological grading standards.

The model was first evaluated on the RoseBlooming dataset [53], which contains continuous visual recordings of two distinct commercial rose varieties (’Samourai 08’ and ‘Blossom Pink’). This dataset categorizes the growth cycle into two primary stages: rose_small (buds and early opening) and rose_large (fully unfolded). Consisting of over 7,000 instances, it serves as an ideal benchmark for testing intra-species variety adaptation.

As detailed in **Table 12**, Flora-YOLO demonstrates consistent performance gains over the YOLO11n baseline. In the challenging rose_small category—which is characterized by minimal pixel areas—the model achieved an improvement of 2.5 percentage points in mAP@0.5 and 2.0 percentage points in mAP@0.5:0.95. This significant gain confirms that our structural optimizations for small-target feature amplification effectively generalize across different floral cultivars.

**Table 12:** Performance comparison on the Rose Blooming dataset.

Model	Category	mAP@.50 (%)↑	mAP@.50:.95 (%)↑	GFLOPs↓
YOLO11n	rose_small	71.1	26.0	6.3
	rose_large	95.5	56.9	
	all	83.3	41.5	
Flora-YOLO (ours)	rose_small	73.6 (+2.5)	28.0 (+2.0)	4.5(−1.8)
	rose_large	96.6 (+1.1)	57.6 (+0.7)	
	all	85.1 (+1.8)	42.8 (+1.3)	

GFLOPs denotes model-level computational complexity; parentheses indicate the relative change compared to the baseline.

In addition to the precision improvements, Flora-YOLO reduced the computational load by 28.6% (from 6.3 to 4.5 GFLOPs). These results confirm that the model maintains superior detection efficiency and accuracy across different greenhouse datasets, validating its practical value for large-scale industrial sorting.

To further test the limits of cross-species generalization, we deployed Flora-YOLO on AriAplBud [54], a large-scale orchard dataset comprising 3,600 images with 110,467 annotated apple buds. Unlike generic flower detection, AriAplBud requires fine-grained classification across six continuous growth stages. It presents extreme visual challenges, including dense target clustering and severe branch occlusion—environmental factors that directly parallel the severe occlusion and small-target constraints (Bottlenecks 2 and 3) addressed in this study.

Despite the drastic semantic shift from greenhouse roses to orchard apple buds, Flora-YOLO maintained its performance superiority. As shown in **Table 13**, our model secured a substantial 1.9 percentage point improvement in mAP@0.5 over the native YOLO11n baseline. Crucially, Flora-YOLO established this performance with the lowest parameter count (1.73 M) and computational load (4.5 GFLOPs) among all tested architectures. This resilience demonstrates that the task-specific gating and attention mechanisms successfully capture invariant phenological traits, regardless of the biological species, providing a robust solution for diverse precision agricultural tasks.

**Table 13:** Cross-species generalization test on the public AriAplBud dataset.

Model	mAP@.5 (%)↑	Params(M)↓	GFLOPs↓
YOLOv3-tiny	76.5	12.13	18.9
YOLOv5n	78.3	2.50	7.1
YOLOv8n	78.7	2.14	6.0
YOLO11n	81.2	2.58	6.3
Flora-YOLO(Ours)	83.1	1.73	4.5

### 4.5. robustness to specific agricultural challenges

As established in the introduction, unstructured foliage occlusion and extreme scale variations (particularly of early-stage buds) represent the primary obstacles in automated greenhouse floriculture. To verify that the proposed architectural innovations explicitly mitigate these challenges, we conducted targeted evaluations using specifically curated extreme-condition subsets.

#### 4.5.1. Performance under severe occlusion.

In high-density greenhouse cultivation, blooming flowers are frequently obscured by overlapping foliage and stems, which disrupts feature extraction. To evaluate the model’s resilience against such structural interference (Bottleneck 2), we constructed an Occlusion Challenge Set featuring scenarios where 20% to 60% of the target’s pixel area is obscured, with the results detailed in **Table 14**.

**Table 14:** Performance comparison under severe occlusion scenarios.

Model	mAP@.5 (%)↑	mAP@.5:.95 (%)↑
YOLOv3-tiny	75.2	43.3
YOLOv5n	76.9	44.6
YOLOv8n	76.7	44.2
YOLO11n	79.1	45.8
Flora-YOLO(Ours)	82.8	48.2

The loss of visual cues penalized all evaluated architectures; however, Flora-YOLO exhibited superior resilience. It achieved an 82.8% mAP@0.5, outperforming the native YOLO11n baseline by 3.7 percentage points. This robustness is primarily attributed to the integration of the ADown adaptive downsampling module and the C3k2_SGB structure. Unlike traditional strided convolutions that often discard fragmented floral textures during spatial reduction, the dual-branch strategy of ADown effectively preserves high-frequency spatial cues that remain visible despite occlusion. Consequently, even when the primary floral center is hidden, the network can leverage peripheral petal edges—amplified by the SGB units—to achieve precise localization.

#### 4.5.2. Evaluation on extremely small object detection.

Detecting extremely small targets, predominantly Degree 0 unbloomed buds (Bottleneck 3), is challenging due to their limited pixel footprint and subtle semantic signatures. We constructed a Small Object Challenge Set consisting of 50 images where the relative bounding box area of targets is less than 3% of the total image area, as presented in **Table 15**.

**Table 15:** Performance comparison for extremely small object detection.

Model	mAP@.5 (%)↑	mAP@.5:.95 (%)↑
YOLOv3-tiny	75.6	43.7
YOLOv5n	78.3	46.1
YOLOv8n	77.7	44.5
YOLO11n	80.8	46.6
Flora-YOLO(Ours)	84.5	48.8

Flora-YOLO significantly outperformed the generic baselines in extreme small-scale recognition. By achieving an 84.5% mAP@0.5, our model established a 3.7 percentage point improvement over the YOLO11n baseline. This precision is a direct consequence of the C2VGA hybrid attention mechanism and the FSIoU loss function. By employing variance-gated channel re-weighting within C2VGA, the model actively identifies information density, amplifying the weak semantic signals of minute buds while suppressing background foliage noise. Furthermore, the inner-focusing dynamic of the FSIoU loss provides more stable gradient guidance for small bounding box regression, minimizing coordinate drift even when visual information is extremely sparse.

### 4.6. Edge device deployment

#### 4.6.1. Android application deployment.

To achieve real-time, on-device flower identification on mobile devices, we deployed the Flora-YOLO model as an Android application. The core of the technical pipeline involved converting and optimizing the best.pt model, trained using the PyTorch framework, to make it suitable for the mobile computing environment.

Firstly, the  .pt model was converted to the ONNX (Open Neural Network Exchange) format to achieve interoperability between frameworks. During this process, to enhance inference efficiency and reduce resource consumption on mobile devices, an FP16 (half-precision floating-point) precision conversion was performed. This operation reduced the model weight data width from 32 bits to 16 bits, significantly decreasing model size and memory footprint. It also effectively utilized mobile GPU hardware that supports half-precision calculations to accelerate inference, all while keeping the loss in detection accuracy within an acceptable range.

Subsequently, the optimized ONNX model was converted to the NCNN format. This conversion process parsed the model into the network structure definition file (.param) and model weight file (.bin) required by NCNN. The resulting NCNN model eliminated the dependency on large deep learning training frameworks, laying the foundation for efficiently running YOLO detection algorithms on resource-constrained mobile devices.

At the application implementation level, the uni-app framework was used to develop the front-end user interface, and the C++-based NCNN inference engine was integrated into the Android project via native plugin technology. After completing all functional development, the project was compiled and packaged to generate a standard Android application package (APK). This APK integrates the user interface, local inference capabilities, and model files, forming a fully self-contained flower grading application.

**Fig 13:**
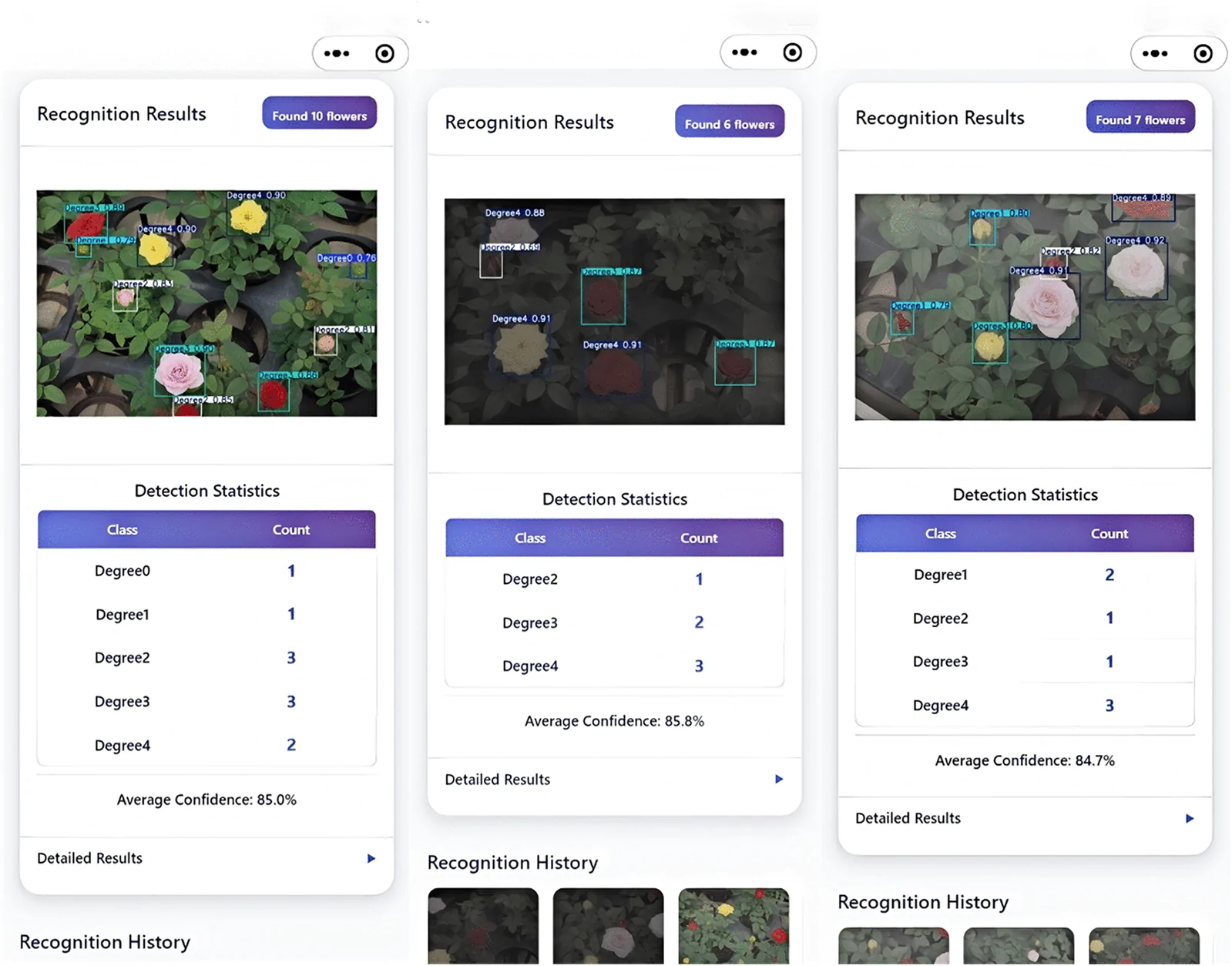
Real-time detection results of the Android application under different environmental conditions (sunny, overcast, and low-light).

To verify the model’s robustness and deployment stability in real-world agricultural environments, we evaluated the optimized Flora-YOLO model (converted to NCNN format for mobile edge execution) on a dedicated held-out test set consisting of independent images across variable field conditions. The evaluation scenarios simulated sunny (sufficient direct light), overcast (diffuse natural light), and low-light (early morning/dusk) greenhouse environments. Rather than relying solely on average confidence indicators, we conducted a quantitative performance evaluation on the deployed terminal. On the held-out test set, the deployed Android application achieved high accuracy with an overall mAP@0.5 of 86.2%, a Precision of 85.8%, and a Recall of 83.4%. Compared to the FP32 GPU baseline model (86.7% mAP@0.5), the mobile-quantized model exhibited a marginal precision drop of less than 0.5%, proving that edge deployment causes negligible accuracy degradation. As shown in Fig 13, the application maintains precise target localization and phenological stage classification across sunny, overcast, and low-light conditions, demonstrating strong environmental adaptability for practical mobile flower grading.

#### 4.6.2. Embedded development board deployment.

To further evaluate the performance and efficiency of the Flora-YOLO model in a true edge computing environment, we selected a development board based on the NXP i.MX 8M Plus SoC as the deployment platform. This platform is particularly suited for such tasks due to its integrated Neural Processing Unit (NPU), which provides up to 2.3 TOPS of hardware acceleration for quantized neural network models.

The deployment on the NXP i.MX 8M Plus is specifically designed for a target application scenario: an automatic grading system integrated onto a mobile agricultural guide rail. In this setup, the detection unit travels along crop rows to perform real-time phenology assessment and quality grading. Unlike static monitoring stations, the mobility of the rail system makes long-distance video cabling impractical, and relying on wireless transmission to a remote server introduces unpredictable latency. Therefore, an embedded edge computing architecture is essential. It enables the mobile unit to process images locally and output grading decisions instantly (within ~90 ms), ensuring high operational efficiency and reliable synchronization with the rail’s spatial positioning system without dependency on external network infrastructure.

The core of the deployment process lay in model format conversion and optimization to adapt to the embedded platform’s runtime environment and hardware accelerator. First, we converted the optimal weight file (.pt format) trained with the PyTorch framework into the ONNX format. As an open intermediate representation, ONNX effectively decouples the model from its specific training framework, facilitating subsequent conversions. To ensure the model could be successfully deployed on the target board, we made necessary adjustments to intermediate layers in the model structure to address the hardware memory limitations of the i.MX 8M Plus.

Subsequently, we utilized the TensorFlow Lite conversion tool to transform the ONNX model into the TensorFlow Lite FlatBuffer (.tflite) format. In this critical step, we performed full integer quantization (INT8), converting the model weights and activations from floating-point numbers to 8-bit integer precision. We utilized a representative calibration dataset of 100 images to determine the dynamic range of activations, ensuring minimal precision loss. This quantization process was vital not only because it significantly reduced the model file size and memory footprint, but more importantly, because it allowed the model to fully leverage the NPU’s hardware acceleration capabilities, as the unit is optimized for low-precision integer operations.

The final INT8 quantized  .tflite model was deployed to the development board using NXP’s eIQ machine learning software development environment and executed inference on the NPU. This achieved efficient flower phenology detection on a resource-constrained edge device.

**Fig 14:**
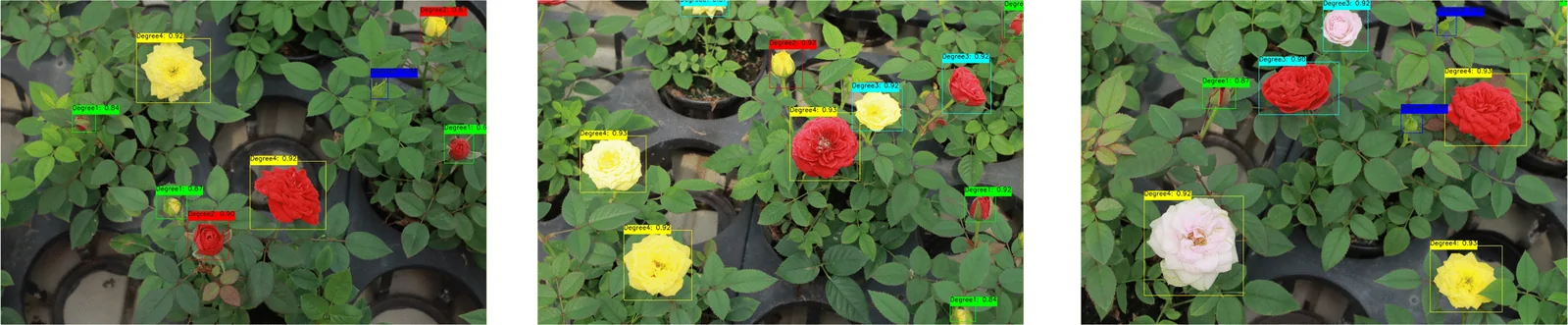
Sample detection results generated by Flora-YOLO running on the NXP i.MX 8M Plus embedded board.

Test results showed that the model could effectively perform the flower phenology detection task on the edge device, as visually demonstrated in Fig 14. In the processing of 100 sample images, the average confidence for valid detection results (confidence > 0.5) reached 0.8774, demonstrating that the model maintained high detection accuracy on this platform. Under this configuration, the model’s average inference speed was 11.05 FPS, with a latency of approximately 90.5 ms. In the context of the mobile agricultural guide rail scenario, this throughput allows for a detection approximately every 9 cm at a travel speed of 1.0 m/s. This high sampling density ensures that each flower (typically spaced 15–30 cm apart) is captured and processed multiple times, meeting the stringent real-time synchronization requirements between the visual recognition output and the rail’s spatial positioning system. These results verify the practical feasibility and effectiveness of deploying Flora-YOLO on resource-limited embedded hardware for automated, on-site floriculture operations.

### 4.7. Discussion

#### 4.7.1. Mechanism of performance enhancement.

To elucidate the internal mechanisms driving the overall performance improvement and verify the effective mitigation of the four primary bottlenecks, we conducted a granular, per-class analysis across the five blooming stages. As demonstrated in Table 16, Flora-YOLO avoids overfitting to a single dominant category; instead, it achieves a synergistic enhancement across the entire phenological spectrum. This granular data confirms that the architectural optimizations provide targeted benefits for specific morphological and environmental challenges.

**Table 16:** Fine-grained performance comparison of YOLO11n and Flora-YOLO across different blooming stages.

Model	Class	mAP@.50 (%)	mAP@.50:.95 (%)
YOLO11n	Degree0	75.8	33.6
	Degree1	86.3	45.7
	Degree2	90.2	56.0
	Degree3	74.9	48.2
	Degree4	90.8	62.8
	All	83.3	48.8
Flora-YOLO (ours)	Degree0	81.3(+5.5)	35.7(+2.1)
	Degree1	87.9(+1.6)	46.9(+1.2)
	Degree2	91.6(+1.4)	56.5(+0.5)
	Degree3	77.2(+2.3)	49.3(+1.1)
	Degree4	91.5(+0.7)	63.1(+0.3)
	All	86.7(+3.4)	50.4(+1.6)

The most substantial performance leap occurs in Degree 0 (bud), which addresses the small-target detection challenge (Bottleneck 3). Traditionally compromised by minimal pixel footprints and visual blending with green sepals, this category saw a substantial improvement of 5.5 percentage points in mAP@0.5. This gain validates that the C2VGA hybrid attention mechanism, through its variance-gated re-weighting, effectively amplifies weak early-stage semantic signals, while the ADown module successfully preserves critical spatial features that are typically lost during conventional downsampling.

Conversely, Degree 2 (partially opened) achieved the highest absolute precision (91.6% mAP@0.5). Morphologically, this stage features distinct petal structures and a stable aspect ratio, representing an optimal feature-extraction state. By integrating the C3k2_SGB module, the network’s capacity to extract these fine-grained textures (Bottleneck 1) is significantly enhanced, resulting in more stable feature representations.

The data also reveals a meaningful distinction between Degree 1 and Degree 3, illustrating the interplay between classification confidence and localization precision. While Degree 1 achieves higher mAP@0.5, Degree 3 surpasses it under the stricter mAP@0.5:0.95 metric (49.3% vs. 46.9%). Degree 1 flowers possess distinct classification features but exhibit jagged, irregular contours that penalize high-IoU regression. The introduction of the FSIoU loss function provides superior shape-aware gradient guidance, addressing Bottleneck 4 (Irregular Morphology) and enabling the model to generate tighter bounding boxes despite significant morphological deformations.

Finally, the lower mAP@0.5 of Degree 3 (77.2%) compared to Degree 0 (81.3%) is rooted in severe inter-class ambiguity and intra-class variance. As a dynamic transitional state, Degree 3 shares overlapping traits with both adjacent stages, featuring complex cross-shadows and extensive self-occlusion (Bottleneck 2). Despite these biological complexities, Flora-YOLO still secured a 2.3 percentage point improvement for this category, demonstrating robust discriminative power in ambiguous states. Ultimately, these results confirm that Flora-YOLO functions as a series of targeted structural interventions, successfully mitigating feature loss and optimizing regression for the inherent challenges of greenhouse environments.

#### 4.7.2. Error analysis and failure modes.

Despite the performance gains of Flora-YOLO, an analysis of failure cases—specifically misclassifications and missed detections—reveals three main practical limitations in real-world applications:

First, grading ambiguity often occurs due to the continuous nature of flower growth and varying camera viewpoints. The transition between late-stage buds (Degree 0) and early opening (Degree 1) is biological and gradual, making hard discrete classification difficult. Additionally, a top-down (zenithal) camera angle can alter the visible shape of the petals compared to a side (lateral) view, occasionally leading to stage misjudgments. This highlights the inherent limitation of using 2D geometric features to classify 3D biological changes.

Second, physical crowding causes missed detections due to Non-Maximum Suppression (NMS) constraints. In densely clustered areas, the bounding boxes of multiple adjacent flowers heavily overlap. If this overlap exceeds the predefined NMS threshold, valid targets are incorrectly suppressed as redundant predictions, leading to false negatives (Bottleneck 2). While the FSIoU loss improves bounding box tightness, distinguishing highly overlapped targets in 2D space remains a standard limitation of current bounding-box regression architectures.

Finally, extreme lighting conditions in greenhouses can reduce feature reliability. Direct sunlight, deep shadows, or specular reflections on wet leaves can alter the visual appearance of floral textures. These illumination changes can disrupt the attention maps of the C2VGA module, weakening the fine-grained features needed for correct classification. This indicates that while the model handles standard greenhouse noise well, vision-only frameworks are still vulnerable to severe, localized illumination shifts.

#### 4.7.3. real-world deployment implications.

The deployment of Flora-YOLO on the NXP i.MX 8M Plus edge platform, achieving an inference speed of 11.05 FPS, demonstrates the practical feasibility of a real-time, edge-oriented grading framework. However, the transition from laboratory benchmarks to greenhouse deployment introduces environmental and operational variables. For instance, automated mobile rail systems inherently introduce motion blur, requiring the model to maintain robust feature extraction within continuous, dynamic video streams.

In this context, the lightweight architecture of Flora-YOLO (4.5 GFLOPs) provides dual operational advantages. First, it ensures a processing latency of under 100 ms, which is a stringent prerequisite for the synchronized operation of mechanical sorting arrays. Second, and perhaps most critical for greenhouse-deployed hardware, the reduced computational load significantly minimizes the thermal dissipation requirements of the edge Neural Processing Unit (NPU). In the high-temperature and high-humidity environments typical of modern greenhouses, preventing thermal throttling is as essential as maintaining detection precision. By operating within a minimal computational envelope, Flora-YOLO provides a vital thermal safety margin, ensuring reliable, continuous operation without hardware degradation or unexpected latency spikes.

#### 4.7.4. Limitations and future directions.

While Flora-YOLO provides a robust and efficient solution for automated flower grading, certain limitations regarding dataset diversity and sensor modalities must be explicitly acknowledged to comprehensively evaluate its current deployment scope. First, the primary training dataset exhibits inherent hardware and environmental constraints. Comprising 1,002 images collected from a single greenhouse facility in Dongying, Shandong Province, the current dataset lacks: (1) Geographic diversity, as environmental conditions are localized to a single facility; (2) Lighting diversity, being restricted primarily to sunny and overcast conditions within the greenhouse structure; (3) Cultivar diversity, as the training images exclusively focus on a single rose cultivar (’Juice Balcony’); and (4) Camera diversity, given that image acquisition relied solely on standard smartphone camera sensors. While our out-of-domain evaluation on public agricultural datasets demonstrated promising zero-shot transferability, systematic dataset expansion across broader geographical regions, diverse lighting scenarios, multiple commercial cultivars, and industrial imaging hardware remains necessary for universal commercial adoption. Second, the current framework relies exclusively on spatial features derived from static 2D frames, which precludes the use of temporal continuity inherent in continuous agricultural monitoring. This reliance can lead to intermittent detection failures, particularly when targets are momentarily obscured by moving greenhouse infrastructure or overlapping foliage during camera traversal (Bottleneck 2).

To systematically address these constraints, future research will pursue three primary avenues. First, we will construct a multi-center, multi-cultivar dataset captured across varied lighting and sensing hardware to enhance domain robustness. Second, we intend to integrate spatio-temporal features by transitioning the framework from static object detection to video-based multi-object tracking (MOT). By leveraging multi-frame consistency, the system can naturally resolve transient occlusion issues—where a target obscured in one frame becomes identifiable as the camera perspective shifts. Third, we plan to explore extreme quantization techniques, such as Post-Training Quantization (PTQ) to INT4 or INT2 precision, enabling deployment on ultra-low-power microcontrollers (e.g., the ESP32 series) to reduce hardware costs for decentralized, large-scale grading nodes. Finally, we envision a transition toward multi-modal phenology monitoring by fusing visual data with depth information (RGB-D) and environmental sensor readings to forecast blooming trends and crop yields in precision floriculture.

## 5. Conclusion

This study presented Flora-YOLO, a lightweight, efficient, and robust object detection framework tailored for the challenges of automated rose phenology grading in complex agricultural environments. By integrating four core architectural optimizations—the C3k2_SGB structure, the ADown adaptive downsampling module, the C2VGA hybrid attention mechanism, and the FSIoU loss function—into the YOLO11n baseline, the proposed model successfully mitigates the four primary agricultural bottlenecks identified in this research: (1) fine-grained morphological ambiguity, (2) severe physical occlusion, (3) extreme small-target detection, and (4) environmental domain shift.

Comprehensive empirical evaluations and rigorous statistical validations demonstrate that Flora-YOLO consistently outperforms state-of-the-art detection models across the primary benchmark dataset, severe domain-shifted scenarios, and multiple public agricultural datasets (encompassing both cross-varietal and cross-species tests). Specifically, Flora-YOLO establishes a new performance-efficiency Pareto frontier, achieving superior precision while maintaining the lowest parameter count (1.73 M) and computational load (4.5 GFLOPs) among all evaluated lightweight variants.

Furthermore, the successful deployment on the NXP i.MX 8M Plus embedded edge platform, achieving a real-time inference speed of 11.05 FPS, demonstrates the framework’s industrial viability. The model’s minimal computational footprint not only ensures high-speed operation on resource-constrained hardware but also provides a vital thermal safety margin for continuous greenhouse deployment. Ultimately, this research offers a statistically sound and highly reliable monitoring solution for the precision floriculture industry, providing a robust architectural template for future AI-driven applications in high-density, unstructured agricultural settings.
